# Supplementary material for: VEGFR-1/Flt-1 inhibition increases angiogenesis and improves muscle function in a mouse model of Duchenne muscular dystrophy
Source: Mol Ther Methods Clin Dev. 2021 Mar 23;21:369–81. doi: 10.1016/j.omtm.2021.03.013 (PMC8055526; doi:10.1016/j.omtm.2021.03.013)
Supplement: Document S1. Supplemental materials and methods, Table S1, and Figures S1–S4 [file mmc1.pdf]

OMTM, Volume *21*

Supplemental information

VEGFR-1/Flt-1 inhibition increases angiogenesis and improves muscle function in a mouse model of Duchenne muscular dystrophy

Jennifer Bosco, Zhiwei Zhou, Sofie Gabriëls, Mayank Verma, Nan Liu, Brian K. Miller, Sheng Gu, Dianna M. Lundberg, Yan Huang, Eilish Brown, Serene Josiah, Muthuraman Meiyappan, Matthew J. Traylor, Nancy Chen, Atsushi Asakura, Natalie De Jonge, Christophe Blanchetot, Hans de Haard, Heather S. Duffy, and Dennis Keefe

1. Antibody Purification

In order to obtain pure antibody (Ab) samples for animal experiments, Chinese hamster ovary (CHO) Epstein-Barr nuclear antigen-1 (EBNA) cells were electroporated with anti-human Flt-1 full IgG lead candidates using the MaxCyte STX system (MaxCyte, Gaithersburg, MD). Two hundred µg/mL anti-human Flt-1 ATUM vector pD2610-v5 DNA and 50 µg/mL XBP1s DNA were electroporated into CHO EBNA cells using MaxCyte 2.1 (10 mL electroporation volume with 400×10^9 cells). After transfection, cells were transferred to a 1-L flask with EX-Cell Advanced CHO Fed-batch Medium (Sigma-Aldrich) for recovery at 37°C. At 24 h after transfection, 3% EX-CELL Advanced CHO Feed 1 (with glucose) (AF1; Sigma-Aldrich, St. Louis, MO) was added and cells were transferred to 33°C. At 48 h after transfection, 3% AF1 and 0.125% N,N-dimethylacetamide were added; 3% AF1 was continued for 10 to 14 days or until viability dropped below 75%. Culture media was collected and frozen at –80°C for purification.

For purification, culture media was thawed and loaded onto an equilibrated MabSelect SuRe column (GE Healthcare Life Sciences) at 20 mg/mL resin. The column was washed with 5 volumes of phosphate-buffered saline (PBS) pH 7. 2 (Invitrogen) and then eluted with 10 volumes of 100 mM glycine pH 3 (Sigma) and finally neutralized with a 50:1 ratio of 1 M Tris hydrochloride (HCl) pH 9. Fractions were pooled and dialyzed overnight at 4ºC in 40 mM Tris HCl pH 8.1 and loaded onto an equilibrated HiTrap Q HP column (GE Healthcare Life Sciences) at 100 mg/mL resin. The column was washed with 10 volumes of 40 mm Tris HCl pH 8.1 and then eluted with 5 volumes of PBS, 1 M sodium chloride. Fractions were pooled and dialyzed into PBS.

2. Pharmacokinetics of 21B3 and 27H6 in Mice, Rats, and Monkeys

Measurement of 21B3 in Mice

Male CD-1 mice (n = 27, Charles River Laboratories, Wilmington, MA) received a single intravenous (i.v.) bolus dose of 10 mg/kg [^{125}I]-21B3. At 0.083, 0.25, and 4 h, and 5, 21, and 28 days after dosing, a blood sample was collected from 3 mice per dose group. At 7 time points after dosing (0.5, 1, 8, and 24 h, and 3, 7, and 14 days), 3 mice per dose group were euthanized and blood, diaphragm, and tibialis samples were collected. Serum samples were precipitated with trichloroacetic acid and centrifuged to precipitate serum proteins; the supernatant fractions and pellets were analyzed. All serum and muscle samples were analyzed for total radioactivity using gamma counting techniques.

Measurement of 27H6 in Mice

Male CD1 mice (n = 42 mice per dose group) received a single i.v. bolus dose of 0.3, 3, or 30 mg/kg [^{125}I]-27H6. At 14 time points after dosing (5, 15, and 30 min; 1, 2, 4, 8, and 24 h; and 3, 5, 7, 14, 18, and 28 days) a terminal blood sample was collected from 3 mice per dose group. To determine the fraction of [^{125}I] that was unbound to 27H6 in each sample, serum samples were precipitated with trichloroacetic acid and centrifuged to precipitate proteins; the supernatant fraction and pellet were analyzed for total radioactivity using gamma counting techniques.

Measurement of 27H6 in Rats

Male Sprague Dawley rats (n = 24 rats per dose group, Charles River Laboratories) received a single i.v. bolus dose of 0.3, 3, or 30 mg/kg 27H6 h(D/A) LALA. Before dose and at 16 time points after dosing (5, 15, and 30 min; 1, 2, 4, 8, and 24 h; and 3, 5, 7, 10, 14, 18, 24, and 28 days), serial blood samples were collected from 3 rats per dose group. 27H6 h(D/A) LALA was measured using an enzyme-linked immunosorbent assay (ELISA).

Measurement of 27H6 in Monkeys

Three dose groups, each consisting of 1 male and 1 female cynomolgus monkey (Covance), received a single i.v. bolus of 0.3, 3, or 30 mg/kg 27H6. Before dose and at 16 time points after dosing (5 min; 1, 2, 6, and 24 h; and 2, 3, 4, 5, 7, 10, 14, 21, 28, 35, and 45 days), serial blood samples were collected and analyzed using an anti–sFlt-1 detection ELISA.

3. Histopathology Analysis

All immunohistochemistry staining was performed on 5 µM paraffin-embedded sections of the diaphragm or tibialis muscles. For CD31 staining, goat anti-CD31/PECAM-1 Ab (R&D Systems, AF3628, 1:50) was used as the primary Ab and isotype IgG as a negative control; biotin-labeled rabbit anti-goat IgG (Vector Laboratories) and ABC Kit (Vector Laboratories) were applied as the detection system and the positive signal was revealed with 3,3′-diaminobenzidine. For detection of fibrosis, rabbit anti-collagen I Ab (Boster Bio, PA2140-2, 1:1000) was used.

BOND Polymer Refine kit (Leica Biosystems, DS9800) was applied as the detection system. The positive cells were identified as brown in color and nuclei were stained blue. The stained slides were scanned with Aperio AT2 scanner (Leica Biosystems). The whole digital slides were viewed and analyzed by Aperio ImageScope (Leica Biosystems). The positive pixel count algorithm was selected and adjusted to cover each individual positive staining for analysis. The data were presented as positivity, which was obtained from the following formula: positivity (%) = positive area (pixels) / total stain area (pixels) × 100%.

4. Measurement of Muscle Perfusion and Muscle Function

Red blood cell flux was measured using a laser Doppler flowmeter with an MP7a probe as previously described,[1] according to the manufacturer's instructions (Moor Instruments). Fur from the right hind leg was removed using a chemical depilatory. Flux was measured at ≥ 10 different spots on the tibialis anterior muscle. An arbitrary unit was determined as the average arbitrary unit value during a plateau phase of each measurement.

Microbubble angiography was performed using the Vevo 2100 Imaging System with Vevo MicroMarker contrast agent (Fujifilm). Mice were sedated, shaved, and depilated over the medial hind limb. Mice were sedated using 4% isoflurane and secured to a heated platform using tape. Pre-warmed ultrasound gel was applied to the probe and the muscle was visualized using B-mode and Doppler ultrasound to locate the femoral artery/vein; the region of interest of exact same size was drawn for the muscle around the vessels. Contrast agent (60 µL containing 2×10^9 bubbles/mL) was administered over 2 s retro-orbitally and non-linear contrast imaging was performed. The peak enhancement was measured using Vevo 2100 software (Fujifilm).

To measure contraction strength, mice were anesthetized with a cocktail of fentanyl citrate (10 mg/kg body weight), droperidol (0.2 mg/kg body weight), and diazepam (5 mg/kg body weight). The left hind limb of each mouse was shaved and aseptically prepared, and each mouse was positioned on a heated platform with its left foot placed on a metal foot plate attached to the shaft of a servomotor. Two platinum electrodes (model E2-12, Grass Technologies) were inserted subcutaneously on either side of the peroneal nerve. A stimulator and stimulus isolation unit stimulated the peroneal nerve via the platinum electrodes to induce a contraction of the anterior crural muscles. For whole muscle contraction, the anterior crural muscles of untreated and 21B3-treated mice were stimulated and the contraction elicited was recorded. The current was adjusted to elicit the maximum twitch and adjusted to 15% additional current for the isometric contraction. The parameters for stimulation were set at a 200 ms contraction duration consisting of 0.5 ms square-wave pulses at 250 Hz. Torque was normalized according to body weight.

The forelimb grip strength test was performed as previously described.[2] Mice were gently pulled by the tail after forelimb-grasping a metal bar attached to a force transducer (Columbus Instruments). Grip strength tests were performed by the same blinded examiner. Five consecutive grip strength tests were recorded, then the mice were returned to the cage for a resting period of 20 min. Three series of pulls were then performed, each followed by a 20-min resting period. The average of the three highest values of the 15 collected values was normalized to the body weight for comparison.

Table S1. Antibody binding to VEGFR-1, VEGFR-2, and VEGFR-3
Example binding affinity of mAbs (50 μg/mL single injection) was measured using a Biacore method (direct coating antigen). Binding to VEGFR-2 and VEGFR-3 was not observed, even at high doses of antibody, indicating that there is no cross-reactivity to these isoforms.

mAb	VEGFR-1	VEGFR-2	VEGFR-3
Blank	2.91	3.64	4.45
13B4	**92.6**	2.75	3
21C6	**181**	1.74	3.25
21B3	**78.7**	3.32	4.26

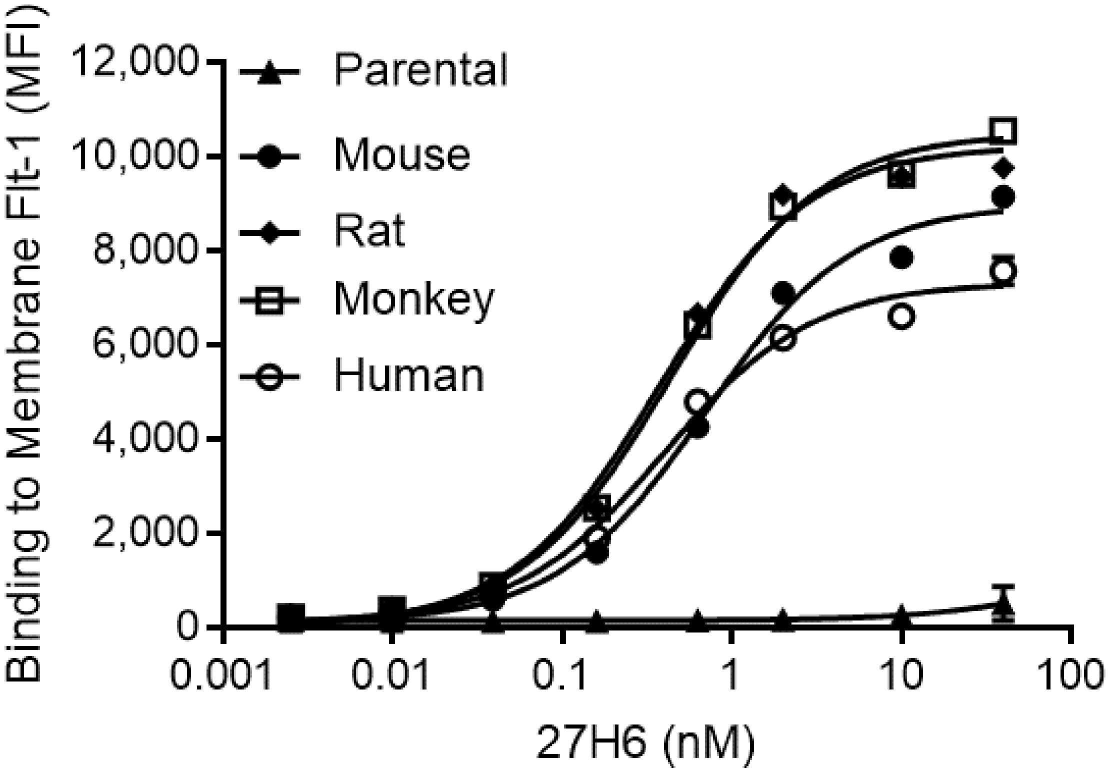

Figure S1. AF647-Labeled 27H6 Binding to Membrane-Bound Flt-1 in HEK293 Cells Overexpressing Mouse, Rat, Monkey, and Human sFlt-1

To assess species-specific binding, 27H6 and a control antibody (Ab) (ZMAB – Human IgG1, AB Biosciences, Concord, MA) were labelled with AF467 (Invitrogen, Waltham, MA) and various concentrations were added to HEK293 cells (ATCC) overexpressing species-specific sFlt-1 (n = 2 for each species). 250,000 cells were plated and incubated overnight. 1 µL of various concentrations of 27H6-AF647 or control antibody was added to the wells, followed by a 15-min incubation at 4ºC. The cells were washed and fixed in 2% paraformaldehyde for 10 min at 4ºC. The samples were analyzed using a FACSCanto II flow cytometer (BD Biosciences, San Jose, CA). Untransfected cells were used as the parental control. MFI, mean fluorescence intensity.

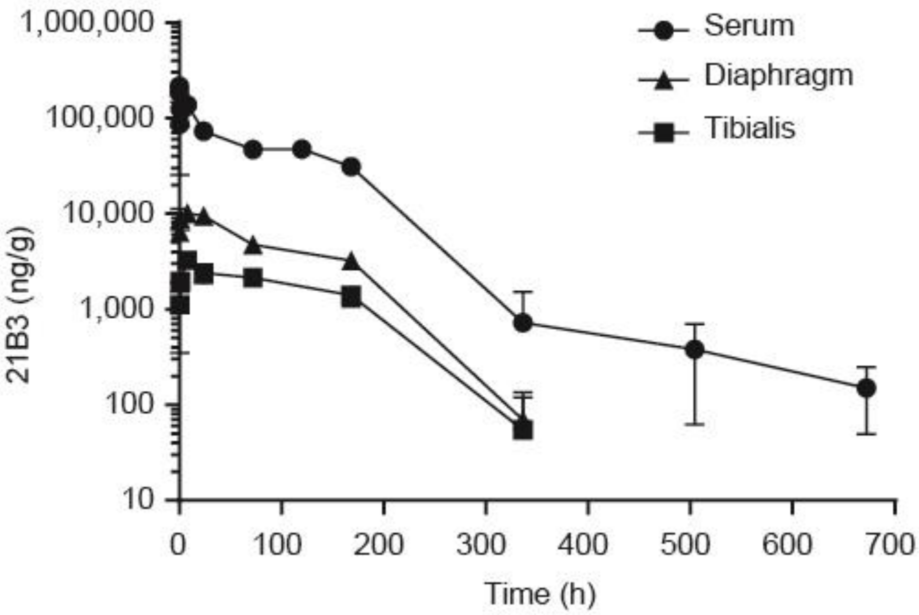

Figure S2. Mean Concentrations of [125I]-21B3 in Serum and in Diaphragm and Tibialis Anterior Muscles of Mice

Mice were administered a single 10 mg/kg intravenous bolus dose of [^{125}I]-21B3 in the serum and skeletal muscles. Concentration-time profiles show mean ± standard deviation of n = 3 mice at each time point. Values represent test article equivalents.

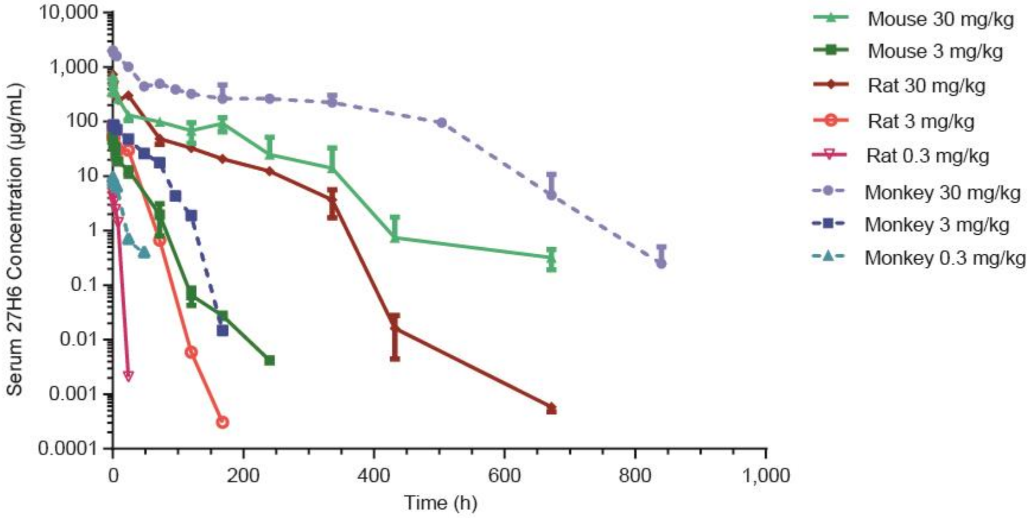

Figure S3. Mean Serum Antibody Concentrations in Mice, Rats, and Monkeys After a Single Intravenous Bolus Injection of 27H6

Rats and cynomolgus monkeys were administered a single intravenous bolus dose of 0.3, 3, or 30 mg/kg 27H6. Blood samples were collected up to 28 days after dosing. Levels of 27H6 were measured using an anti–Flt-1 detection enzyme-linked immunosorbent assay. Concentration-time profiles show mean ± standard deviation of n = 3 mice, n = 2 rats, and n = 2 monkeys at each time point. There was a non-parallel terminal clearance slope across different dose levels (0.3 to 30 mg/kg) with higher clearance at lower doses. Target-mediated disposition occurred when drug exposure was lower than target Flt-1 concentrations.

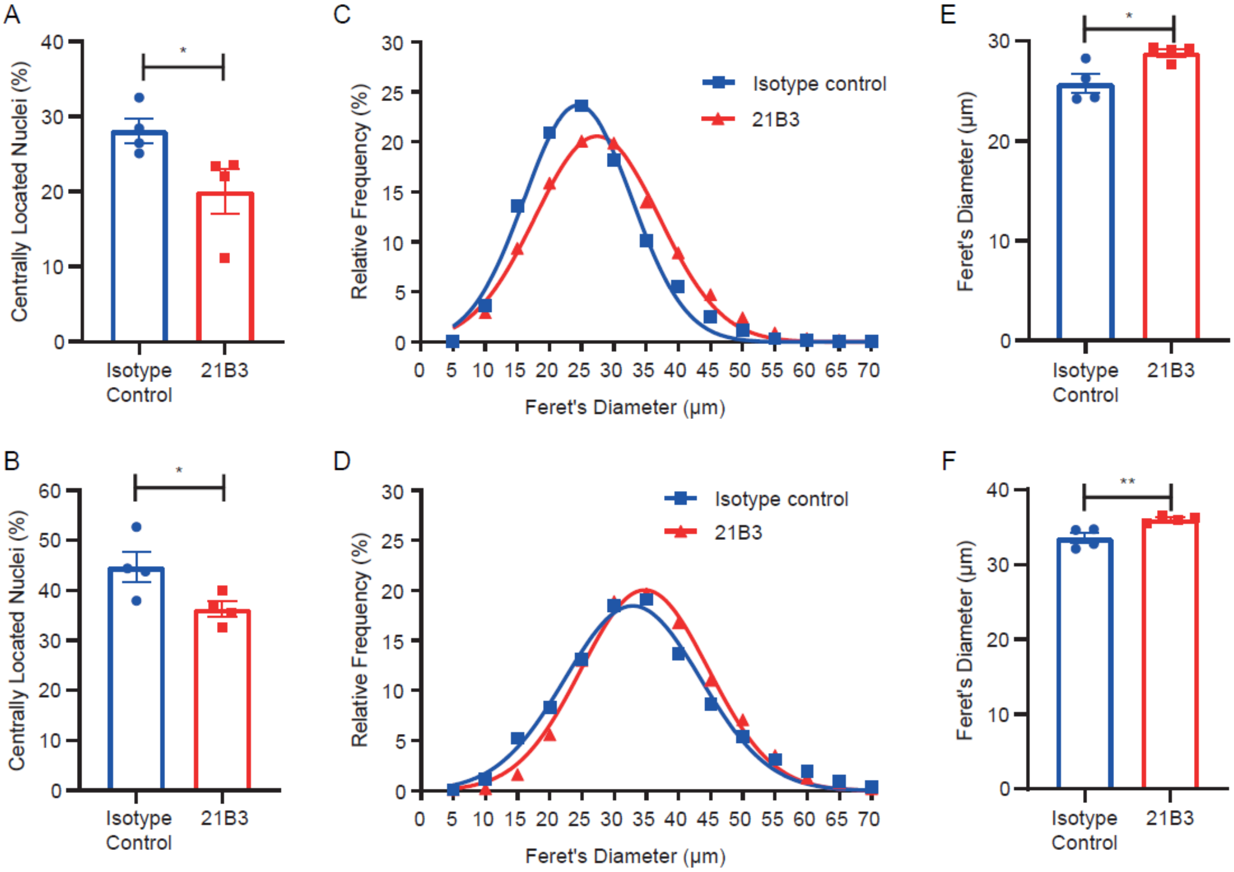

Figure S4. Administration of 21B3 improves muscle histology and function in mdx mice.
(A, B) Diaphragm and tibialis anterior muscle fiber turnover is reduced in *mdx* mice administrated 20 mg/kg 21B3 twice weekly for 4 weeks compared with controls (n = 4 per dose group) as evaluated by centrally located nuclei. (C, D) Distributions and (E, F) averages of mean fiber diameter in diaphragm and tibialis anterior muscle of *mdx* mice administrated 21B3 or control. *$p < 0.05$, **$p < 0.01$ using t tests.

Histological analysis of diaphragm (A, C, E) and tibialis anterior (B, D, F) muscle of *mdx* mice (9–10 weeks old) administered 20 mg/kg 21B3 i.v. twice weekly for 4 weeks. Tissues were frozen fresh using LiN_2 chilled isopentane and stored at –80ºC. Transverse cryosections (8 µm thick) were used for all histological analyses. Hematoxylin & eosin staining were performed as previously described[3]. Microscopic images were captured by a DP-1 digital camera attached to a BX51 fluorescence microscope with 10 × or 40 × UPlanFL N objectives (all from Olympus). Image processing and manual enumeration of the fiber diameter was conducted using Fiji.[4]

References

1. Verma, M, Asakura, Y, Hirai, H, Watanabe, S, Tastad, C, Fong, GH, Ema, M, Call, JA, Lowe, DA, and Asakura, A (2010). *Flt-1* haploinsufficiency ameliorates muscular dystrophy phenotype by developmentally increased vasculature in *mdx* mice. *Hum Mol Genet* **19**: 4145-4159.

2. Aartsma-Rus, A, and van Putten, M (2014). Assessing functional performance in the *mdx* mouse model. *J Vis Exp* **85**: 51303.

3. Verma, M, Shimizu-Motohashi, Y, Asakura, Y, Ennen, JP, Bosco, J, Zhou, Z, Fong, GH, Josiah, S, Keefe, D, and Asakura, A (2019). Inhibition of FLT1 ameliorates muscular dystrophy phenotype by increased vasculature in a mouse model of Duchenne muscular dystrophy. *PLoS Genet* **15**: e1008468.

4. Schindelin J, Arganda-Carreras I, Frise E, Kaynig V, Longair M, Pietzsch T, Preibisch, S, Rueden, C, Saalfeld, S, Schmid, B, *et al.* (2012) Fiji: an open-source platform for biological-image analysis. *Nat Methods* **9**: 676–682.
