# Supplementary material for: VEGFR-1/Flt-1 inhibition increases angiogenesis and improves muscle function in a mouse model of Duchenne muscular dystrophy
Source: Mol Ther Methods Clin Dev. 2021 Mar 23;21:369–81. doi: 10.1016/j.omtm.2021.03.013 (PMC8055526; doi:10.1016/j.omtm.2021.03.013)
Supplement: Document S2. Article plus supplemental information [file mmc2.pdf]

Molecular Therapy
Methods & Clinical Development
Original Article

VEGFR-1/Flt-1 inhibition increases angiogenesis and improves muscle function in a mouse model of Duchenne muscular dystrophy

Jennifer Bosco,[1] Zhiwei Zhou,[1] Sofie Gabriëls,[2] Mayank Verma,[3] Nan Liu,[1] Brian K. Miller,[1] Sheng Gu,[1] Dianna M. Lundberg,[1] Yan Huang,[1] Eilish Brown,[1] Serene Josiah,[1,4] Muthuraman Meiyappan,[1] Matthew J. Traylor,[1,5] Nancy Chen,[1] Atsushi Asakura,[3] Natalie De Jonge,[2] Christophe Blanchetot,[2] Hans de Haard,[2] Heather S. Duffy,[1] and Dennis Keefe[1,6]

[1]Shire Human Genetic Therapies, a Takeda company, Lexington, MA, USA; [2]argenx BVBA, Zwijnaarde, Belgium; [3]Stem Cell Institute, Paul and Sheila Wellstone Muscular Dystrophy Center, Department of Neurology, University of Minnesota Medical School, Minneapolis, MN, USA

Duchenne muscular dystrophy is characterized by structural degeneration of muscle, which is exacerbated by localized functional ischemia due to loss of nitric oxide synthase-induced vasodilation. Treatment strategies aimed at increasing vascular perfusion have been proposed. Toward this end, we have developed monoclonal antibodies (mAbs) that bind to the vascular endothelial growth factor (VEGF) receptor VEGFR-1 (Flt-1) and its soluble splice variant isoform (sFlt-1) leading to increased levels of free VEGF and proangiogenic signaling. The lead chimeric mAb, 21B3, had high affinity and specificity for both human and mouse sFlt-1 and inhibited VEGF binding to sFlt-1 in a competitive manner. Proof-of-concept studies in the *mdx* mouse model of Duchenne muscular dystrophy showed that intravenous administration of 21B3 led to elevated VEGF levels, increased vascularization and blood flow to muscles, and decreased fibrosis after 6–12 weeks of treatment. Greater muscle strength was also observed after 4 weeks of treatment. A humanized form of the mAb, 27H6, was engineered and demonstrated a comparable pharmacologic effect. Overall, administration of anti-Flt-1 mAbs in *mdx* mice inhibited the VEGF:Flt-1 interaction, promoted angiogenesis, and improved muscle function. These studies suggest a potential therapeutic benefit of Flt-1 inhibition for patients with Duchenne muscular dystrophy.

INTRODUCTION

Duchenne muscular dystrophy (DMD) is an X-linked genetic disorder, primarily affecting males, that is characterized by progressive muscle degeneration and weakness.[1] Patients are usually diagnosed early in childhood and have a life expectancy of up to 20–30 years. DMD results from mutations in the *DMD* gene,[1] which codes for dystrophin, a membrane-associated structural protein that functions within the dystrophin-associated protein complex to stabilize sarcolemma and maintain normal interactions with the local microvasculature.[2] The mutations lead to dysfunctional myofibers and subsequent muscle damage, followed by reduced ambulation and as the diaphragm muscle degenerates, loss of ventilation.

The dystrophin-associated protein complex includes neuronal nitric oxide synthase, a key enzyme in the production of the vasodilation signaling molecule, nitric oxide.[3] Lack of nitric oxide results in decreased blood flow in the microvasculature, subsequent functional ischemia, and eventual myofiber necrosis and fibrosis.[4] It has been hypothesized that increased muscle perfusion, for example, through the use of vasoactive agents or by an increase in vascular density, could improve muscle function.[5–7]

Vascular endothelial growth factor (VEGF) is a signaling protein that induces angiogenesis by binding to tyrosine kinase receptors such as Flt-1 (also known as VEGFR-1) and VEGFR-2 on the surface of endothelial cells.[8] There is evidence that modulation of VEGF signaling is a potential therapeutic option in DMD,[9,10] as seen in a number of animal models in which increased VEGF levels were associated with angiogenesis and reduced muscle injury.[11] However, efficacy appears to be limited following administration of VEGF alone,[12] and toxicities such as vascular leak and disorganized angiogenesis have been observed following VEGF administration.[13,14] Additionally, *in vivo* studies have shown that exogenous VEGF has a short half-life ($t_{1/2}$) of approximately 30 min, in part, due to its binding to Flt-1.[15]

Flt-1 is considered to be a negative regulator of angiogenesis because it binds VEGF with higher affinity than VEGFR-2 but has lower kinase activity and thus acts as a VEGF "sink." In addition, Flt-1 is expressed as both a membrane-bound receptor in tissues and as an alternatively spliced soluble protein lacking the transmembrane

Received 21 August 2020; accepted 17 March 2021;
https://doi.org/10.1016/j.omtm.2021.03.013.

[4]Present address: LifelineBio, Somerville, MA, USA
[5]Present address: Mosaic Biosciences, Boulder, CO, USA
[6]Present address: Stealth BioTherapeutics, Newton, MA, USA
Correspondence: Jennifer Bosco, Shire Human Genetic Therapies, Cambridge, MA, USA.
E-mail: jennifer.bosco@takeda.com

This is an open access article under the CC BY-NC-ND license (http://creativecommons.org/licenses/by-nc-nd/4.0/).

domain in blood (sFlt-1).[16] sFlt-1, but not membrane-bound Flt-1, is selectively expressed in hypoxia[17] and pre-eclampsia,[18] suggesting that it plays a prominent role in the regulation of VEGF-induced angiogenesis. Therapeutics that inhibit Flt-1 may be efficacious in promoting angiogenesis and restoring oxygenation to damaged muscles in patients with DMD.[5] Indeed, loss of Flt-1 has been shown to improve angiogenesis and ameliorate muscle weakness in *mdx* mice, which lack dystrophin and are widely used as a model of DMD.[19,20] Although the DMD phenotype in *mdx* mice is less severe than in humans, the mice show muscle degeneration, decreased vasculature, extensive fibrosis, and other key characteristics of the disease.[21]

To evaluate the potential of Flt-1 antagonism as a therapeutic strategy in DMD, we used phage display techniques to develop a monoclonal antibody (mAb) directed against Flt-1 and engineered it to enhance its affinity, specificity, and potency. The mAb, designated 21B3, inhibited Flt-1, decreased levels of free sFlt-1, increased levels of free VEGF in serum, and improved muscle function in *mdx* mice through an increase in vascularization and perfusion. Our findings support the link between Flt-1 antagonism and muscle perfusion and function and indicate that inhibition of Flt-1 may provide potential therapeutic benefits to patients with DMD.

RESULTS

Discovery and characterization of lead mAb 21B3

To obtain antibodies that target Flt-1, phage libraries comprising the conventional antibodies (i.e., consisting of heavy and light chains) derived from llamas immunized with a human sFlt-1 construct were initially screened for binding to sFlt-1.[22] In addition to conventional antibodies, camelids also produce functional Abs that lack light chains, thus providing numerous advantages in therapeutic mAb development.[23,24] Antigen-binding fragments (Fabs) of interest were engineered as chimeric llama-human immunoglobulin Gs (IgGs) composed of llama variable heavy (VH)-chain/variable light (VL)-chain and human constant regions.[25] The selected Fabs were screened for binding affinity with both human and mouse sFlt-1 orthologs, potency in inhibiting VEGF binding, selectivity for sFlt-1 compared with VEGFR-2 and VEGFR-3 (Table S1), and cross-species reactivity (Figure S1). Chain shuffling was performed on an early lead candidate to obtain variants with higher affinity for sFlt-1. One resulting variant with improved affinity for sFlt-1, mAb 21B3, was reformatted to a human IgG1 Fc backbone (27H6) and further evaluated. 21B3 was also engineered to a construct with a mouse IgG1 Fc backbone for use in proof-of-concept studies in *mdx* mice.

To characterize our lead mAb, we first employed surface plasmon resonance-based binding assays that measured the interaction of 21B3 with immobilized recombinant human and murine sFlt-1. 21B3 exhibited equivalently high affinity for both mouse sFlt-1 and human sFlt-1 (KD^{obs} 1.4×10^{-10} M versus 0.7×10^{-10} M, respectively; Figures 1A and 1B). Competition enzyme-linked immunosorbent assay (ELISA) experiments over a range of 169 pg/mL to 10 μg/mL 21B3 further revealed that the 21B3 interaction prevented the binding of VEGF to sFlt-1 in a dose-dependent manner, with comparable half-maximal inhibitory concentration (IC_{50}) values of 5.6 ng/mL (1.24×10^{-10} M) and 7.4 ng/mL (1.64×10^{-10} M) for the mouse and human orthologs, respectively (Figures 1C and 1D). The functional consequence of sFlt-1-mediated VEGF sequestration was assessed in cell culture experiments. As expected, activation of VEGFR-2 by VEGF in human umbilical vein endothelial cells (HUVECs; which express the Flt-1 receptor), as indicated by VEGFR-2 phosphorylation, was minimal in the presence of sFlt-1, owing to high-affinity binding of VEGF to sFlt-1. However, the addition of 21B3 inhibited both mouse and human sFlt-1 sequestration of VEGF and consequently rescued VEGFR-2 phosphorylation to 85% of maximum ($p < 0.0001$ for both compared with sFlt-1 alone by two-way ANOVA; Figure 1E).

In addition to evaluating the binding activity of 21B3, hydrogen-deuterium exchange mass spectrometry was used to identify the binding epitope of sFlt-1. Two peptide sequences that exhibited significant inhibition of deuterium exchange upon 21B3 binding were mapped to the Flt-1 domain 2 (D2), a region of Flt-1 known to be critical for VEGF binding (Figure 1F), demonstrating that bound mAb directly competes with VEGF for binding to sFlt-1. Given that these interactions are spatially distant from the membrane-spanning residues of full-length VEGFR-1/Flt-1, such competitive binding is expected to apply to the membrane-anchored Flt-1 isoform as well.[26]

Pharmacokinetics and pharmacodynamics of anti-Flt-1 mAb (21B3) in mice

The pharmacokinetic profile of 21B3 was investigated following a single 10-mg/kg intravenous (i.v.) bolus dose of [^{125}I]-21B3 in CD-1 mice. An additional study using non-radioactive 21B3 was also completed, with similar results (data not shown). A target-mediated disposition profile was observed in serum (Figure S2), with a terminal elimination $t_{1/2}$ of 34 h (Table 1). 21B3 was detected in diaphragm and tibialis anterior muscle up to 14 days after administration, with higher exposure in the diaphragm compared with the tibialis anterior.

To corroborate the *in vitro* findings on the antagonistic effects of 21B3 and to evaluate the durability of the effects, *mdx* mice were injected with 1, 3, or 10 mg/kg i.v. bolus 21B3 twice weekly for up to 12 weeks. Prior exploratory studies have shown the highest efficacy with twice-weekly dosing (data not shown). 21B3 levels increased in a dose- and time-dependent manner over the first 7 weeks and were sustained for the remaining 5 weeks (Figure 2A). A dose-dependent decrease in free sFlt-1 levels was observed compared with control, likely as a result of binding with 21B3. The decrease was significant at 2 weeks for the 10-mg/kg dose ($p < 0.0001$, 2-way ANOVA; Figure 2B); by 4 weeks, the extent of decrease was similar for all three doses ($p < 0.05$ at least for all). As expected, the reduction in free sFlt-1 was associated with a significant increase in free serum VEGF versus control ($p < 0.0001$ for the 10-mg/kg dose at 2 weeks, and $p < 0.0001$ for the 3- and 10-mg/kg doses at 6 weeks, 2-way ANOVA; Figure 2C).

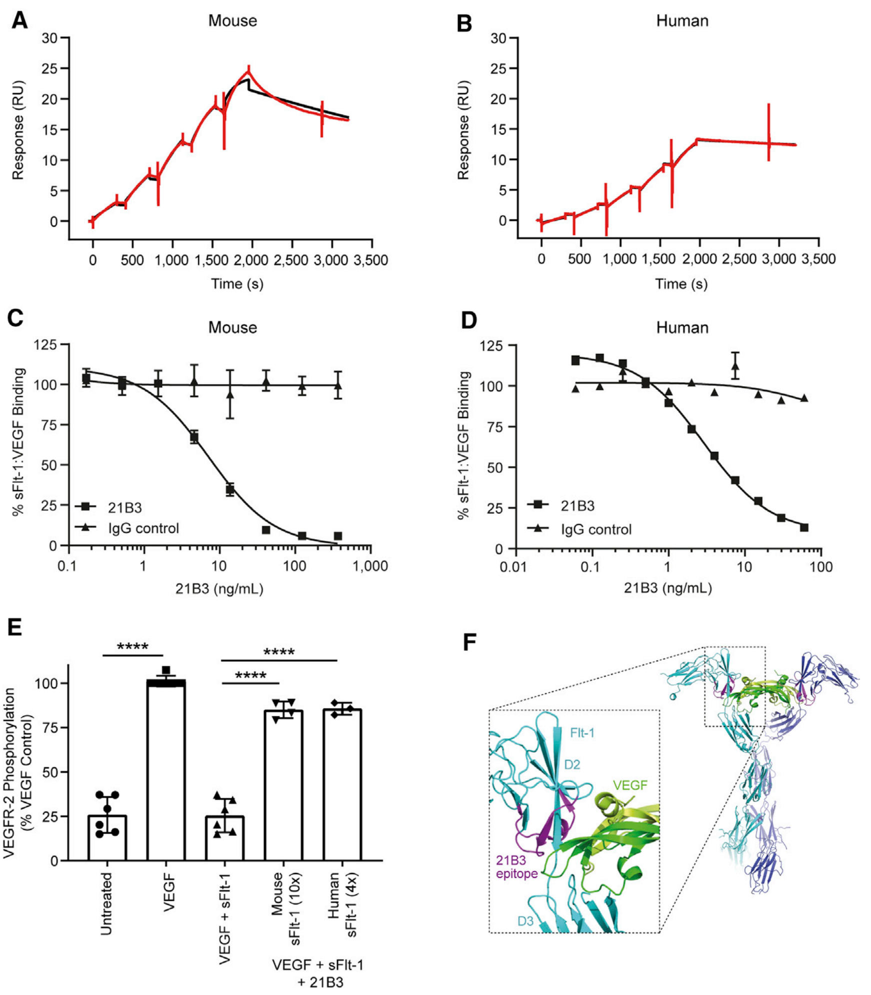

Figure 1. Characterization of 21B3

(A and B) Representative curves from the Biacore affinity analysis of 21B3 binding to (A) mouse or (B) human Flt-1. The observed response for the kinetic titration series is shown in red, and the fit to a 1:1 interaction model is shown in black. (C and D) Competition ELISA of VEGF binding to (C) mouse (n = 2 representative images) or (D) human sFlt-1 (n = 3) in the presence of increasing concentrations of 21B3. Data are presented as mean and standard deviation. (E) Inhibition of VEGFR-2 phosphorylation by mouse and human sFlt-1 in HUVECs. The VEGF bar represents 100% of the phospho-VEGFR2 signal. The data thus represent a percent of the signal seen with VEGF alone; for example, the addition of sFlt-1 inhibited the signal to ~25% compared with VEGF alone. Phosphorylation increased with the addition of 10-fold molar excess mouse 21B3 and 4-fold excess human 21B3 compared with sFlt-1 (n = 3–6; data represent mean and standard deviation). ****$p < 0.0001$ by ordinary ANOVA with multiple comparisons. (F) Ribbon model of the sFlt-1:VEGF complex (PDB 5T89) highlighting the 21B3 epitope. sFlt-1 is shown in blue and cyan for each member of the complex dimer, and the VEGF dimer is shown as dark and light green. The two peptides in sFlt-1 domain 2 that were shown to interact with 21B3 using hydrogen-deuterium exchange are depicted in magenta. RU, response unit.

Effect of anti-Flt-1 mAb (21B3) on capillary density and fibrosis in *mdx* mice

The impact of 21B3-mediated Flt-1 inhibition on capillary density in diaphragm and tibialis anterior muscle was assessed in *mdx* mice administered 21B3 twice weekly for 6 or 12 weeks. A dose-dependent increase in CD31 positivity, a marker of endothelial cells, was observed in both diaphragm and tibialis anterior muscle after 6 and 12 weeks of 21B3 treatment (Figures 3A and 3B); the increases were statistically significant with higher doses of 21B3 ($p < 0.05$ or better versus control, 2-way ANOVA).

The extent of fibrosis was measured as an indicator of muscle health following treatment with 21B3. There were no changes in levels of collagen type 1 (Col-1), a marker of fibrosis, in the diaphragm compared with control after 6 weeks of 21B3 administration; however, Col-1 levels decreased significantly after 12 weeks at all three dose levels ($p < 0.0005$ or better, 2-way ANOVA; Figure 3C). There were no significant changes in Col-1 levels in the tibialis anterior at any of the dose levels after 6 or 12 weeks, although a slight decrease was seen at 12 weeks in the 3-mg/kg and 10-mg/kg groups (Figure 3D).

Effect of anti-Flt-1 mAb (21B3) on blood flow and muscle strength in *mdx* mice

Functional studies were conducted in *mdx* mice administered 20 mg/kg 21B3 twice weekly for 4 weeks. To determine if the increase in capillary density was associated with increased blood vessel function, we examined changes in blood flow, red blood cell (RBC) flux, and the rate of blood flow (as indicated by blood volume) in the hind legs. Compared with control mice, there was a significant increase in RBC flux and blood volume in mice that received 21B3 ($p < 0.05$, 2-way ANOVA; Figures 4A–4D), indicating the presence of functional blood vessels.

To evaluate whether the increased blood flow induced by 21B3 translated to improvement in muscle strength in *mdx* mice, grip strength and strength of muscle contraction were assessed. Contraction strength of the anterior crural muscles was greater in mice administered 21B3 compared with controls ($p < 0.05$, 2-way ANOVA; Figure 4E). Similarly, initial grip strength was greater in mice administered 21B3 than in controls ($p < 0.0001$, 2-way ANOVA; Figure 4F).

In addition, fiber diameters and centrally nucleated fibers were measured in both tibialis anterior muscle and diaphragm. The number of centrally located nuclei was significantly reduced in mice

Table 1. Pharmacokinetic characteristics of [^{125}I]-21B3 in serum and in diaphragm and tibialis anterior muscle of mice following a single 10-mg/kg i.v. bolus dose

Tissue	AUC_{last}, μg · h/g	AUC_{inf}, μg · h/g	C_{max}, μg/g	t_{max}, h	$t_{1/2}$, h
Serum	11,900	11,900	222	8	34.4
Diaphragm	1,180	1,180	9.7	8	41.5
Tibialis anterior	446	448	3.12	8	44.8

Values for pharmacokinetic parameters represent measurement of test article-derived radioactivity. Mean values are shown (n = 13 mice). AUC_{last}, area under the curve from time 0 to the last measurable concentration; AUC_{inf}, area under the curve from time 0 to infinity; C_{max}, maximum concentration; t_{max}, time when maximum concentration occurred; $t_{1/2}$, half-life.

treated with 21B3 compared with controls in both muscles, indicating increased fiber stability (Figure S4).

Development and characterization of humanized anti-Flt-1 mAb, 27H6

Following the promising effects of 21B3 observed in *mdx* mice, modifications were made to engineer a lead humanized mAb intended for evaluation as a clinical candidate. Eleven amino acid changes were made in the VH and VL framework to humanize 21B3 from llama, five residues were modified in the VH/VL complementarity-determining region (CDR) regions to address potential sequence liabilities (including a potential aspartate isomerization site within the light chain CDR3), and two changes were made in the Fc region of the human IgG1 backbone to reduce effector function (L242A and L243A sequence mutations in the CH2 domain).[27]

The resulting humanized construct, 27H6, was characterized and compared with 21B3. The affinity of 27H6 for human and mouse sFlt-1 (dissociation constant [KD] 0.72×10^{-10} M and 1.06×10^{-10} M, respectively; Figure 5A) was minimally affected by the mutations, and the IC_{50} for inhibition of VEGF binding (5.5 ng/mL [1.22×10^{-10} M]) was similar to that of 21B3. VEGFR-2 phosphorylation in HUVECs was rescued to 68% and 89% of maximum following the addition of 2 times and 4 times molar excess of 27H6, respectively (Figure 5B). In addition, Biacore studies showed that binding to Fcγ receptors required for effector function was severely attenuated (data not shown). Binding of 27H6 was comparable between cell lines overexpressing mouse, rat, cynomolgus monkey, and human Flt-1; 50% effective concentration (EC_{50}) values for each species were similar, indicating suitability for further studies in these species (see Figure S1).

mdx mice were administered 1, 3, or 10 mg/kg 27H6 i.v. twice weekly to assess exposure and pharmacodynamic effect. At high doses, 27H6 levels reached steady state by 6 weeks (Figure 6A). Compared with controls, free sFlt-1 levels in serum decreased in a dose- and time-dependent manner ($p < 0.05$ or better, 2-way ANOVA; Figure 6B), and there was a sustained increase in free VEGF levels ($p < 0.05$ or better, 2-way ANOVA; Figure 6C). Interestingly, the inhibition of sFlt-1 occurred at much lower levels of 27H6 (IC_{50} = 0.2377 nM) than the observed EC_{50} for elevation of free VEGF (EC_{50} = 24.17 nM), suggesting that a significant proportion of VEGF continued to be retained by membrane-bound Flt-1/VEGFR-1, even when the majority of sFlt-1 was bound by the mAb (Figure 6D).

The pharmacokinetic profile of 27H6 was evaluated in mice, rats, and monkeys. Volume of distribution at steady state indicated that tissue distribution was primarily limited to the central compartment (i.e., blood and highly perfused organs; Table 2). Non-linear kinetics were observed in all three species because high doses were associated with lower clearance and, correspondingly, longer $t_{1/2}$ (see Figure S3), likely due to the target-mediated disposition phenomenon.

DISCUSSION

The current standard of care in DMD is treatment with corticosteroids along with supportive care and targeted therapeutic treatment for specific patient subpopulations.[28] Recently approved and in-development gene-based therapies targeting DMD are limited to the small proportion of patients with specific mutations and primarily focus on increasing levels of dystrophin in muscle fibers.[29,30] While some increase in muscle strength is seen with these types of therapy, they do not adequately address the loss of functional sympatholysis associated with dystrophin deficiency, which leads to sustained vasoconstriction, ischemia, and muscle degeneration.[4,31] Instead, studies have suggested that improving angiogenesis may be an effective approach,[5,7] We hypothesized that by modulating VEGF signaling through targeting of Flt-1 and promoting angiogenesis at the initial signaling step of VEGF binding, subsequent events in DMD pathophysiology can be diminished without the overt toxicities encountered with VEGF supplementation therapy. Indeed, increased vascularization and muscle strength have been observed in *mdx* mice to correlate with both developmental and conditional *Flt-1* deficiency.[19,20]

Small interfering RNA (siRNA) technology has been shown to be effective in reducing the expression of sFlt-1 in animals.[32] However, the findings were limited by rapid oligonucleotide clearance, and lack of target specificity is a major limitation for siRNA-based therapeutics.[33] In addition, commercially available mouse antibodies were found to have low affinity for sFlt-1.[20] As an alternative, we used a phage display approach to identify an anti-Flt-1 mAb with longer exposure and engineered it for high target specificity and affinity. Furthermore, we formatted the Fc regions of the mAbs for use in different species during development.

Data from *in vitro* and *in vivo* studies together supported the proposed mechanism of action of the anti-Flt-1 mAbs. As expected, the mAbs competed with VEGF for binding to Flt-1 and prevented the sequestration of free VEGF by the Flt-1 sink. Subsequently, free VEGF levels became elevated and available for binding to VEGFR-2 to initiate angiogenic signaling, as indicated by increased phosphorylation. Another possibility is that increased levels of free VEGF protein had a feed-forward effect on VEGF transcription through

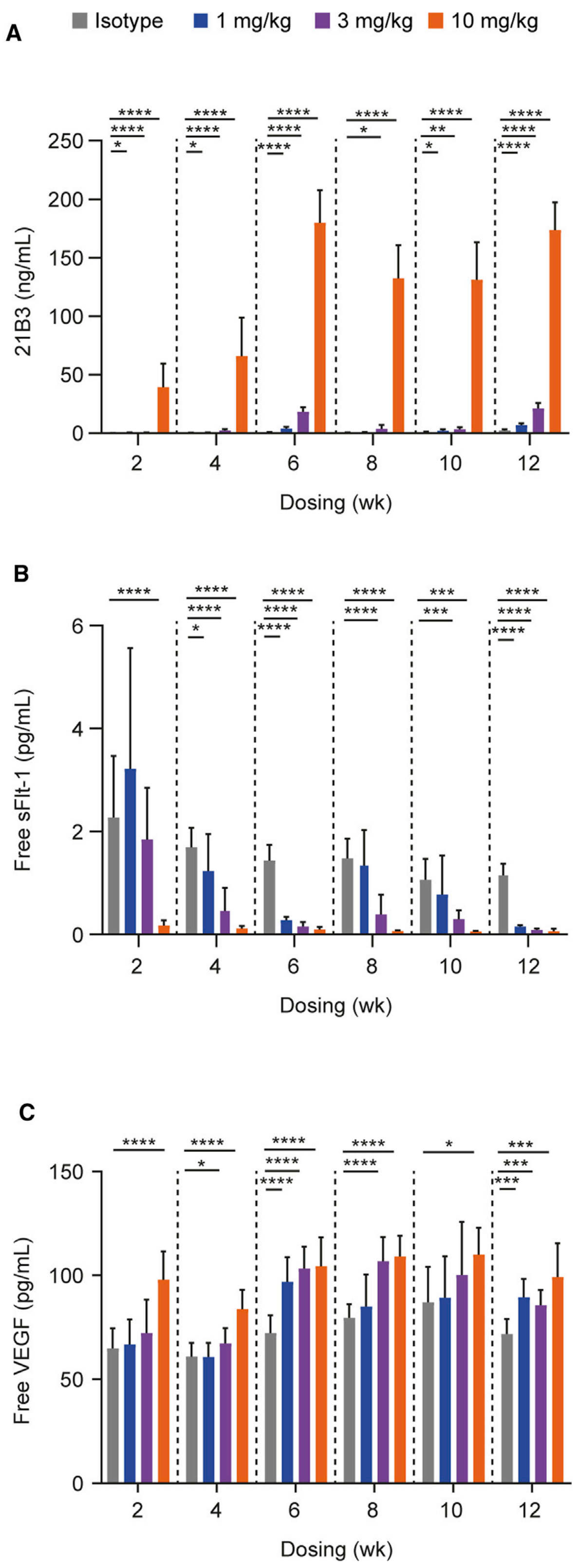

Figure 2. Serum analysis of *mdx* mice treated with 21B3 twice weekly for up to 12 weeks

(A–C) Serum levels of (A) 21B3, (B) free sFlt-1, and (C) free VEGF in *mdx* mice. Data from two studies were combined for the analysis; in one study, mice were treated for up to 6 weeks, and in the other study, mice were treated for up to 12 weeks. In each study, mice were treated with 1, 3, or 10 mg/kg 21B3 (n = 15 each group) or isotype control (n = 10) twice weekly. Samples at 2, 4, 7, and 10 weeks were collected before dosing. Data are presented as mean and standard deviation. *p < 0.05, **p < 0.005, ***p < 0.0005, ****p < 0.0001 by mixed-effects ANOVA.

VEGFR-2.[34] It is unlikely that the increase in free VEGF results from the displacement of VEGF from Flt-1, as VEGF has an extremely high affinity for Flt-1.[5,35] Studies in *mdx* mice further demonstrated proof of concept in that treatment with mAbs increased free VEGF levels, capillary density, muscle perfusion, and strength and also reduced fibrosis. Given that VEGF plays a central role in both angiogenesis and myogenesis,[5,36] the activation of VEGFR-2 by VEGF in muscle tissue likely induced a myogenic response in addition to an angiogenic response. The increased capillary density observed following the administration of 21B3 mAb may also be functioning to increase the stem cell pool by creating more stem cell niches.[37] Further studies in mice to measure mRNA and protein expression in skeletal muscle along with markers of VEGFR-2 signaling would confirm the importance of muscle regeneration as well as angiogenesis in the functional improvements and decreased fibrosis observed.

Levels of free sFlt-1 decreased significantly with mAb treatment compared with isotype; however, we noted that free sFlt-1 levels also decreased in isotype-treated *mdx* mice, which could be attributed to aging of the animals, as suggested by observations in human neonates.[38,39] Studies with the humanized form of the mAb, designated 27H6, suggest comparable pharmacologic characteristics with 21B3, including affinity and inhibitory activity, reduction in free sFlt-1 levels, and increase in free VEGF levels.

Exposure of 21B3 and 27H6 increased with dose in mice, rats, and monkeys in a greater-than-dose-proportional manner, and both mAbs displayed a non-linear clearance profile. Non-linearity is commonly observed for mAbs and in this case likely stemmed from target-mediated clearance. At the highest dose, mAb clearance accelerated when levels dropped below 100 μg/mL in monkeys and ∼10 μg/mL in rodents, consistent with the target-mediated clearance profile of a mAb directed against epidermal growth factor receptor.[40] At these high doses, binding of Flt-1, which is ubiquitously expressed in endothelial cells,[41,42] was at capacity and resulted in higher levels of free Abs and longer serum $t_{1/2}$. At lower doses, the shorter $t_{1/2}$ can be attributed to internalization of mAb complexes with both sFlt-1 and membrane-bound Flt-1. Interestingly, the serum $t_{1/2}$ of both 21B3 (∼35 h) and 27H6 (∼70 h) was unusually short compared with the typical $t_{1/2}$ for IgG mAbs in humans (2–3 weeks)[43] and monkeys (6.5 days),[44] even with the higher doses; this could be attributed to extensive binding to membrane Flt-1, as indicated by our studies in cell lines overexpressing Flt-1. Consequently, the presence of a competing target could potentially exhaust the pool of mAbs, leaving enough uninhibited sFlt-1 to act as a VEGF sink. Despite this

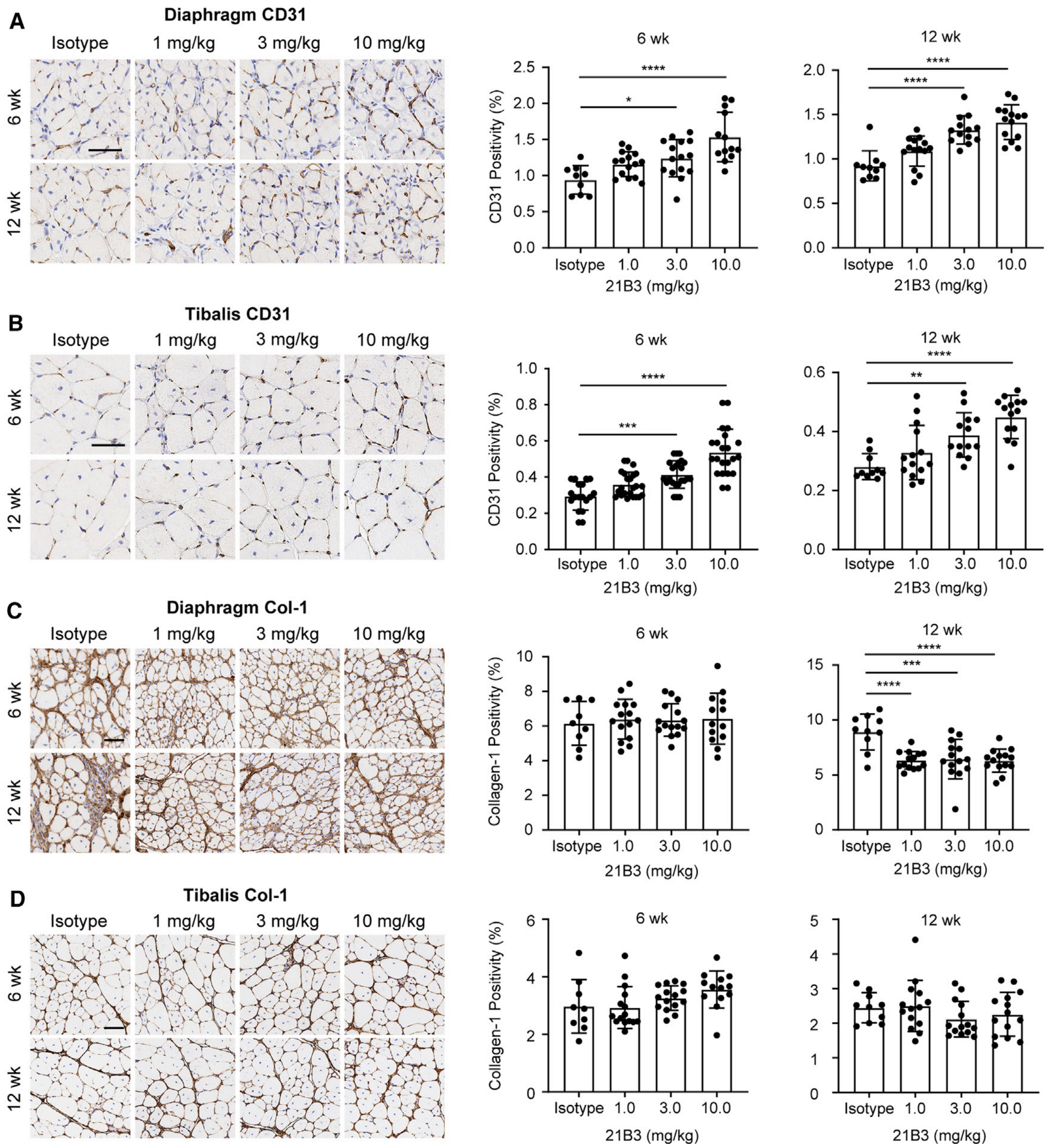

Figure 3. Histological analysis of angiogenesis and muscle pathology in diaphragm and tibialis anterior muscle of *mdx* mice after administration of 21B3
(A–D) Staining for CD31 in (A) diaphragm and (B) tibialis anterior muscle and for Col-1 in (C) diaphragm and (D) tibialis anterior muscle of *mdx* mice treated with control and 1, 3, and 10 mg/kg 21B3 twice weekly for 6 and 12 weeks (n = 9 or more mice per dose group and time point). Bars represent mean and standard deviation. ***p < 0.0005, ****p < 0.0001 by ordinary ANOVA with multiple comparisons.

additional interaction and the higher affinity of VEGF for sFlt-1 (30 pM) compared with the affinity of mAbs (70 pM), our studies show that sFlt-1 is effectively neutralized, and free VEGF levels are elevated at the doses tested. Furthermore, whereas sFlt-1 is known to be a key negative regulator of VEGF-mediated angiogenesis, inactivation of membrane-bound Flt-1 also could have contributed to the pharmacodynamic effects observed *in vivo*. Understanding of the role of membrane-bound Flt-1 and the quantitative relationship among sFlt-1, membrane-bound Flt-1 expression, and mAb exposure across species will be important for predicting the mAb pharmacokinetic profile and dosing strategy during translation to clinical studies.

Modulation of Flt-1 increased vascularization and subsequently improved muscle function in *mdx* mice and thus has the potential for therapeutic benefit in DMD. However, caution should be taken in extrapolating the effects of vascularization in mice to the human

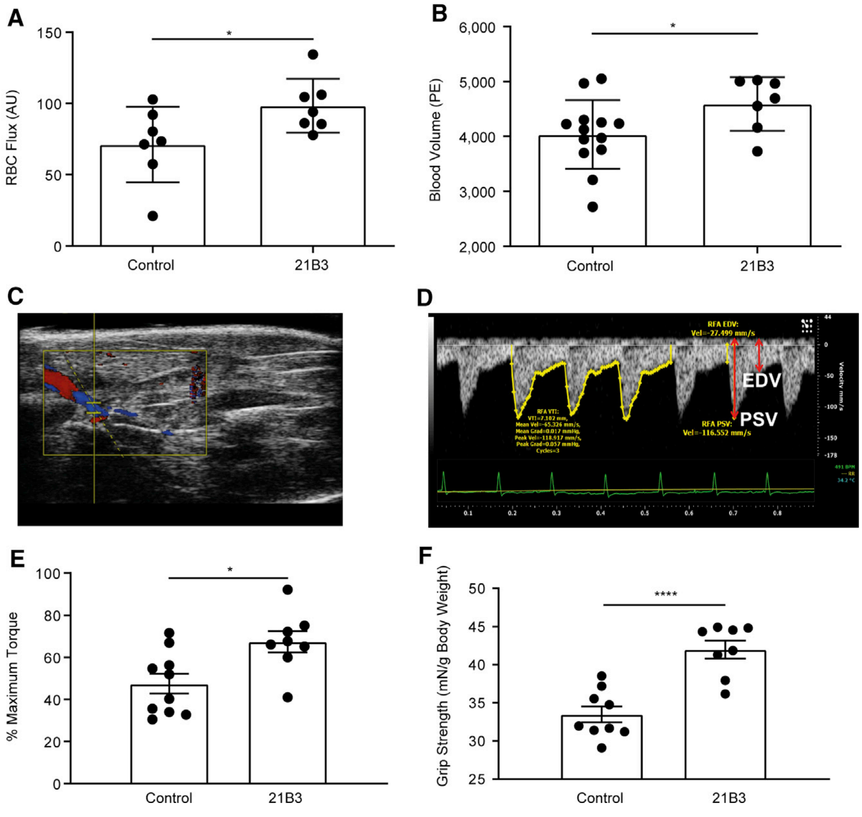

Figure 4. Muscle perfusion and contractile function in *mdx* mice treated with 21B3

(A) RBC flux increased in tibialis anterior muscle of *mdx* mice administered 20 mg/kg 21B3 or control twice weekly for 4 weeks compared with controls (n = 7 mice per dose group). (B) Blood volume, which indicates the rate of blood flow, in the blood vessels of control (n = 13) and 21B3-treated (n = 7) mice, as determined using microbubble angiography. (C and D) Representative image of microbubble angiography-mediated ultrasonography of the femoral artery in thigh muscle of an *mdx* mouse after injection of microbubbles. (E) Contraction strength, as measured by relative torque, in tibialis anterior muscle of control (n = 10) and 21B3-treated mice (n = 8). (F) Grip strength in control (n = 9) and 21B3-treated mice (n = 8); results from the first set of tests are shown. Data represent mean and standard deviation. AU, arbitrary units; mN, millinewtons; PE, peak enhancement. *$p < 0.05$, ****$p < 0.0001$ using t tests.

response. For instance, differences such as the higher capillary density in rodents compared with humans,[45,46] differential VEGFR expression,[47] and molecular and histological differences between human and murine musculature may impact response to treatment.[48]

One of the hallmarks of DMD is fibrosis throughout the muscle, which progresses as a result of repeated cycles of muscle degeneration/impaired regeneration accompanied by inflammation.[49] Increased vascularization is expected to attenuate fibrosis, at least in part, by reducing the hypoxic environment initiated by nitric oxide synthase deficiency. In our studies, increasing vasculature with 21B3 treatment decreased fibrosis to a significant extent in the diaphragm but not in the tibialis anterior. This was not surprising, as there was an approximately three-fold lower level of baseline fibrosis in the tibialis anterior muscle compared with the diaphragm; thus, a significant response would be difficult to detect. Indeed, *mdx* mice present lower levels of fibrosis at 3 weeks old; however, differences could be expected in mouse models with a more severe phenotype in the tibialis anterior such as *mdx*^{5cv} mice.[50,51] In addition, muscle ischemia has been shown to be potentiated in *mdx* mice following exposure to light exercise,[52] and this could be rescued using nitric oxide donors, phosphodiesterase-5 inhibitors, or neuronal nitric oxide synthase gene therapy;[5,53–55] thus, it may be of interest to examine whether the impact of 21B3 on fibrosis and muscle function is more pronounced under these conditions.

Our *in vivo* studies of mAb antagonists against Flt-1 indicate that this is a promising strategy for the treatment of DMD. Improvement in muscle function, particularly in the diaphragm, could not only extend life expectancy but also prevent or delay ambulation loss, thereby enhancing patients' quality of life. However, this approach does not address the underlying cause of the disease, and continuous treatment would be necessary. Thus, further investigation of the effects of long-term modulation of VEGF through this mechanism is needed. Exploratory toxicology studies of a single 3- to 60-mg/kg dose in rats showed a dose-dependent decrease in blood pressure and increase in heart rate, as well as increased cardiac contractility and decreased cardiac relaxation with elevated VEGF levels over 24 h. However, compensation with steady-state elevated VEGF levels remains to be investigated.

An anti-Flt-1 approach could also be used synergistically with other therapeutic approaches to treating DMD. For example, modulation of tissue permeability via VEGF has been shown to improve adeno-associated viral vector efficiency in skeletal and cardiac muscle.[56] Thus, anti-Flt-1 therapy can potentially be used as an adjuvant therapy along with gene therapy for DMD. Anti-myostatin agents that were under development increased muscle cross-sectional area but did not provide functional improvements,[57,58] possibly due to continued ischemia. A combination of anti-Flt1 mAb and anti-myostatin mAb may therefore be beneficial. Increased vascularization may also have potential benefit in other conditions that have an ischemic underpinning due to loss of vasculature. For example, maternal levels of placental-derived sFlt-1 are elevated in pre-eclampsia and may play a role in the development of bronchopulmonary dysplasia in pre-term infants. In rat models of pre-eclampsia and chorioamnionitis, treatment with an anti-Flt-1 mAb improved infant

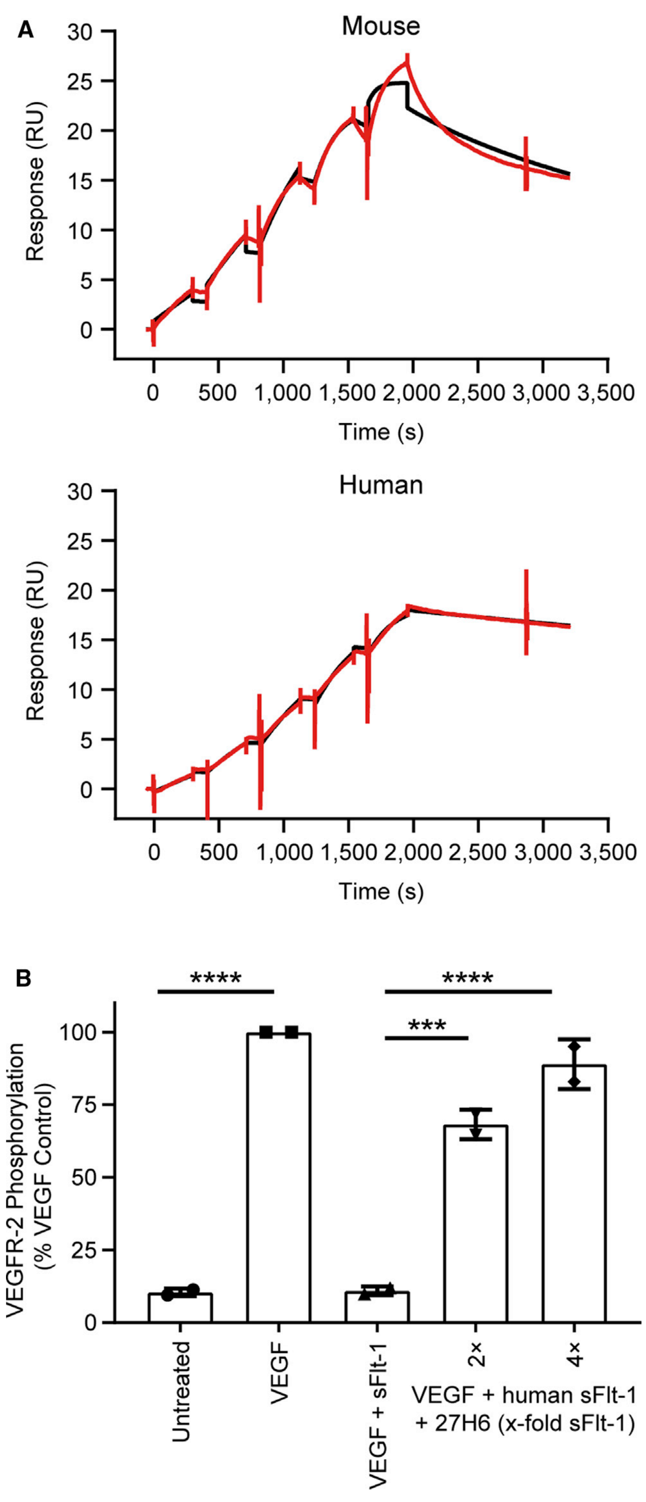

Figure 5. Characterization of humanized 27H6

(A) Representative curves from the Biacore affinity analysis of 27H6 binding to mouse or human sFlt-1. The red curved line shows the observed response; the black curved line shows the fitted response to the kinetic titration series based on a 1:1 stoichiometric interaction. (B) VEGFR-2 phosphorylation in the presence of human sFlt-1 and 27H6 in HUVECs (n = 2 representative data shown as mean and standard deviation). The VEGF bar represents 100% of the phospho-VEGFR2 signal. The data represent a percent of the signal seen with VEGF alone. ***p < 0.0005, ****p < 0.0001 by ordinary one-way ANOVA.

lung structure and function, including increased vascular density and alveolarization, and prolonged neonatal survival.[59]

In conclusion, treatment with mAb antagonists of Flt-1 effectively promoted angiogenesis and improved muscle pathology and *in vivo* muscle function in a mouse model of DMD. Further exploration of this mechanism of action for the treatment of DMD and other conditions associated with high Flt-1 levels is warranted.

MATERIALS AND METHODS

mAb discovery

Abs were developed as previously described.[22] Briefly, llamas were immunized with recombinant human sFlt-1 (Abcam, Cambridge, MA, USA). The VH and VL regions of llama conventional Abs were amplified, and the Fabs were cloned into phage vectors to generate a phage display library. Following one round of phage selection on human sFlt-1 and subsequently two rounds on mouse sFlt-1, human-mouse cross-reactive Fabs were produced in periplasmic fractions of *Escherichia coli* infected with phage particles. Chain shuffling was used to find natural variants with higher affinity for the target. The VH regions of the lead candidates were shuffled back into the VL library, and the clone with the highest affinity was chosen during affinity selections combined with off-rate washes. Abs were expressed in Chinese hamster ovary cells (Sigma-Aldrich, St. Louis, MO, USA) and purified as described in Supplemental materials and methods, 1.

Surface plasmon resonance binding assay for sFlt-1 Ab binding

To identify a mAb that could be evaluated in both pre-clinical animal studies and in clinical trials, a surface plasmon resonance-binding assay with single cycle kinetics (Biacore; GE Healthcare Life Sciences, Marlborough, MA, USA) was used for measuring binding affinity of mAbs to human and mouse sFlt-1. Recombinant mouse sFlt-1 (R&D Systems, Minneapolis, MN, USA) or human sFlt-1 (Abcam) were immobilized to a CM5 series S sensor chip (GE Healthcare Life Sciences). Five mAb concentrations ranging from 0 to 5.0 nM were analyzed for each sample. For each dilution, a 5-min association step was followed by a 40-min dissociation step in mobile phase buffer. The chip surface was regenerated using glycine (pH 2.0) buffer at 60 μL/min with 30 s stability.

Measurement of anti-sFlt-1 mAbs, free sFlt-1, and free VEGF

Anti-Flt-1 Fab levels were measured using a standard ELISA assay. Plates were coated with 1 μg/mL mouse Flt-1 (human Fc; R&D Systems) or human sFlt-1 (Abcam). After blocking, anti-Flt-1 Fabs from screening selections were added to the plate for 1 h. Fabs were detected using a rabbit anti-c-Myc horseradish peroxidase (HRP)-conjugated Ab (Bethyl, Montgomery, TX, USA). After 1 h, the plate was developed with tetramethylbenzidine substrate, stopped with 0.5 M

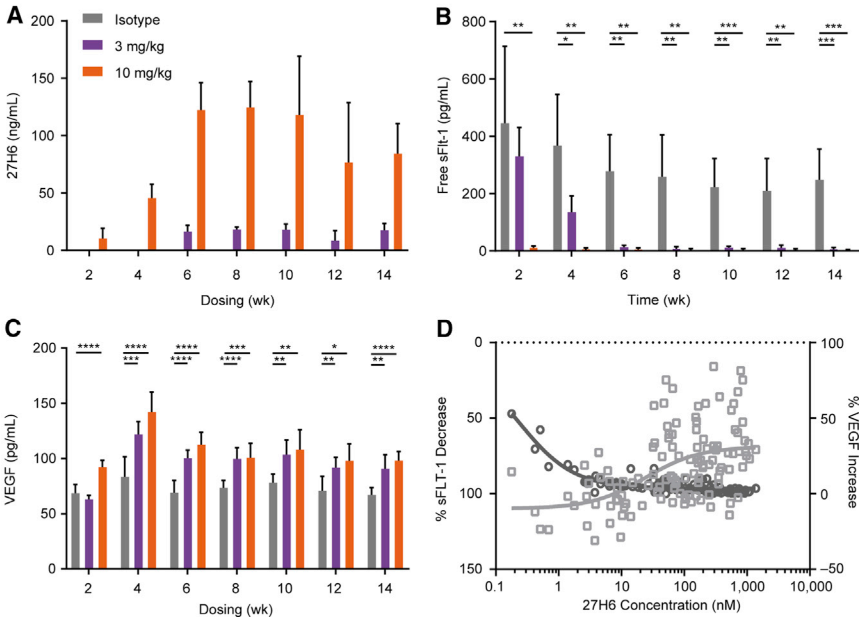

Figure 6. Serum analysis of *mdx* mice treated with humanized 27H6 for 16 weeks

(A) Serum levels of 27H6 increased in a dose- and time-dependent manner (n = 8 for each dose group at each time point). Data represent mean and standard deviation. (B) Free sFlt-1 levels significantly decreased in the presence of 27H6 compared with controls ($p < 0.0001$). (C) VEGF levels increased significantly compared with controls. (D) Relationship between 27H6 concentrations and change in levels of free sFlt-1 and free VEGF in serum of *mdx* mice treated i.v. with 1, 3, and 10 mg/kg 27H6 twice weekly for 6 weeks (n = 8 for each dose group). Percentage increase/decrease represents change compared with levels before treatment. Circles represent change in sFlt-1 levels; squares represent change in VEGF levels. Each point represents an individual mouse. *$p < 0.05$, **$p < 0.005$, ***$p < 0.0005$, ****$p < 0.0001$ by ANOVA with mixed effects.

H_2SO_4, and read at 450 nm (SpectraMax; Molecular Devices, San Jose, CA, USA).

Anti-Flt-1 mAb levels were measured using a standard ELISA assay (Meso Scale Discovery [MSD], Rockville, MD, USA). Plates were coated with 2 μg/mL mouse or human sFlt-1 protein. After blocking, anti-Flt-1 full IgG Abs (21B3 and humanized 27H6) or serum samples from animal studies were added to the plate for 1 h, followed by the addition of 1 μg/mL goat anti-mouse IgG SULFO-TAG Ab (MSD). After 1 h, the plate was developed with MSD Read Buffer (MSD) and read on an MSD Imager 2400 (MSD).

To detect free sFlt-1, ELISA assays were run as described above using plates coated with 2 μg/mL anti-sFlt-1 full IgG lead Ab candidates. After blocking, mouse sFlt-1 protein standard and serum samples were added. 2 μg/mL biotinylated goat anti-mouse sFlt-1 (R&D Systems) was added, followed by 1 μg/mL streptavidin SULFO-TAG Ab (MSD). The plate was developed with MSD Read Buffer.

Free VEGF in diluted serum samples was quantified in a solid-phase sandwich ELISA, as per manufacturer protocol (MMV00; R&D Systems).

sFlt-1 competition assays

Competition ELISA was conducted to measure mAb inhibition of VEGF binding to sFlt-1 using plates coated with 250 ng/mL mouse VEGF (R&D Systems) or human VEGF (R&D Systems) in 0.2 M carbonate-bicarbonate buffer. Anti-sFlt-1 Fabs from screening selections and full IgG candidates (series of dilutions from 20 μg/mL) were added to an equal volume of 20 ng/mL mouse or 80 ng/mL human recombinant VEGFR-1/Flt-1 Fc chimera (R&D Systems) for 1 h and then added to the plate for 30 min. For both competition assays, a mouse anti-histidine TAG biotin-conjugated Ab (Bio-Connect, Huissen, the Netherlands) was added for 1 h, followed by washing and a 1-h incubation with streptavidin-HRP (R&D Systems). The plate was developed for 10–20 min with tetramethylbenzidine substrate, stopped with 0.5 M H_2SO_4, and then read at 450 nm.

Phosphorylation of VEGFR-2

HUVECs were incubated for 10 min with serum-free media containing anti-sFlt-1 candidates, mouse VEGF (100 ng/mL), and mouse sFlt-1 for the mouse assay or anti-Flt-1 candidate, human VEGF (100 ng/mL), and human sFlt-1 for the human assay. Cells were washed and lysed, and phosphorylated VEGFR-2 was measured using an AlphaScreen sandwich immunoassay following the manufacturer's instructions (ALSU-PVGFR-A500; PerkinElmer, Waltham, MA, USA).

Epitope mapping by hydrogen-deuterium exchange

Epitope mapping was performed by hydrogen-deuterium exchange mass spectrometry. Human sFlt-1 (10 μg) or sFlt-1:21B3 mixture (10 μg:20 μg) was incubated in deuterium oxide labeling buffer (50 mM phosphate, 100 mM sodium chloride, pH 7.4) for 0 s, 30 s, 2 min, 10 min, 1 h, or 4 h. Deuterium exchange was quenched by adding 100 μL of buffer containing 3.4 M guanidine hydrochloride and 0.73 M tris-(2-carboxyethyl) phosphine hydrochloride for a final pH of 2.5. Samples were then subjected to pepsin digestion, and the resultant peptides were analyzed using ultra-performance liquid chromatography-tandem mass spectrometry (UPLC-MS/MS; Waters Acquity UPLC instrument [Milford, MA, USA] coupled to a MicrOTOF-Q II mass spectrometer [Bruker, Billerica, MA, USA]). Peptide identification was performed by searching MS/MS data against the sFlt-1 sequence using Mascot (Matrix Science, Boston, MA, USA). Raw MS data were processed using HDExaminer software (Sierra

Table 2. Pharmacokinetic characteristics of humanized 27H6 in serum of mice, rats, and monkeys following a single i.v. bolus dose

Species	Dose, mg/kg	AUC_{inf}, μg · h/mL	$t_{1/2}$, h	CL, mL/h/kg	V_{ss}, mL/kg
Mouse	3	906	25.7	3.3	69.8
	30	26,137	75.5	1.14	120
Rat	0.3	33.6	1.88	8.94	41.8
	3	1,575	8.46	1.84	33.1
	30	21,017	28.6	1.41	82.6
Monkey	0.3	125	9.89	2.28	25.5
	3	3,360	9.43	0.922	29.9
	30	174,954	37.5	0.117	30.5

Values for pharmacokinetic parameters in rats represent measurement of test article-derived radioactivity. Mean values are shown (n = 42 mice, n = 24 rats, and n = 2 monkeys per dose group). CL, clearance; V_{ss}, steady-state volume of distribution.

Analytics, Modesto, CA, USA). Deuterium levels were calculated using the average mass difference between the deuterated peptide and its native form (t_0).

Pharmacokinetics and pharmacodynamics of anti-sFlt-1 mAbs in mice, rats, and monkeys

To measure 21B3 concentrations in serum and tissues, CD-1 mice (8 weeks old) were administered a single i.v. bolus dose of 10 mg/kg [^{125}I]-21B3, and blood samples were collected up to 28 days after dosing for analysis of serum mAb concentrations. Diaphragm and tibialis anterior muscle tissue was collected up to 14 days after dosing. All serum and tissue samples were analyzed for total radioactivity, and data are reported as test article equivalents (see Supplemental materials and methods, 2).

To measure 27H6 concentrations, CD-1 mice were administered a single i.v. bolus dose of 0.3, 3, or 30 mg/kg 27H6. Rats (8 weeks old) and monkeys (2–3 years old) were administered the same doses of humanized 27H6 formatted to human IgG1 containing LALA mutations in the Fc. Blood and muscle samples were collected up to 28 days after dosing in mice and rats, and blood samples were collected up to 45 days after dosing in monkeys. 27H6 levels were measured in mice, rats, and monkeys using an ELISA (see Supplemental materials and methods, 2).

3-week-old male *mdx* mice (C57BL/10ScSn-*Dmd*^{mdx}; Jackson Laboratory, Bar Harbor, ME, USA) were administered 1, 3, or 10 mg/kg 21B3, 27H6, or isotype control i.v. twice weekly. At this age, muscle necrosis and weakness begin in the diaphragm, whereas the mice are also weaned and can accommodate repeated i.v. injections. Wild-type controls were not included in this study, as no correction of disease phenotype was expected in a non-disease model, and the underlying cause of the disease was not being targeted. Blood samples from 21B3-treated mice were collected up to 6 or 12 weeks, and samples from 27H6-treated mice were collected up to 12 weeks to measure levels of mAb, free Flt-1, and free VEGF (see Supplemental materials and methods, 2).

Pharmacokinetic parameters were calculated using non-compartmental analyses with Phoenix WinNonlin version 6.3 (Certara, Princeton, NJ, USA).

Histopathology analysis

All immunohistochemistry staining was performed on 5 μM paraffin sections of diaphragm or tibialis anterior muscle from *mdx* mice (3 weeks old) treated with 1, 3, or 10 mg/kg 27H6 i.v. twice weekly for 6 or 12 weeks. In agreement with the literature,[7,21] findings from a natural history study showed that peak/plateau fibrosis and necrosis in limb muscle and diaphragm occurred between 6 and 12 weeks (data not shown), and thus, these time points captured the endpoints before and after maximum levels were reached. For CD31 staining, goat anti-CD31/PECAM-1 Ab (R&D Systems; AF3628, 1:50) was used as the primary Ab and isotype IgG as a negative control; biotin-labeled rabbit anti-goat IgG (Vector Laboratories, Burlingame, CA, USA) and ABC Kit (Vector Laboratories) were applied as the detection system and the positive signal were revealed with 3,3′-diaminobenzidine. For detection of fibrosis, rabbit anti-Col-1 Ab (Boster Bio, Pleasanton, CA, USA; PA2140-2, 1:1,000) was used, and the BOND Polymer Refine kit (Leica Biosystems, Buffalo Grove, IL, USA; DS9800) was applied as the detection system. The positive cells were identified as brown in color, and nuclei were stained blue. The stained slides were scanned with an Aperio AT2 scanner (Leica Biosystems), and the whole digital slides were viewed and analyzed by ImageScope (Leica Biosystems). The positive pixel count algorithm was selected and adjusted to cover each individual positive staining for analysis. The data were presented as positivity, which was obtained from the following formula: positivity (%) = positive area (pixels)/total stain area (pixels) × 100%. See Supplemental materials and methods, 3.

Measurement of muscle perfusion and function

Muscle function analyses were performed on *mdx* mice (9–10 weeks old) administered 20 mg/kg 21B3 i.v. twice weekly for 4 weeks. As opposed to other studies that showed increased capillary density using a dose of up to 10 mg/kg, a higher dose was chosen for these studies to ensure sufficient exposure to the mAb for confirmation of functional changes; this was also based on our evolving understanding of the relatively short $t_{1/2}$ and rapid clearance of 21B3. RBC flux in the tibialis anterior muscle was evaluated using a laser Doppler flowmeter with an MP7a probe as previously described,[19] according to the manufacturer's instructions (Moor Instruments, Wilmington, DE, USA). Microbubble angiography was performed using the Vevo 2100 Imaging System (Fujifilm VisualSonics, Toronto, ON, Canada) with a Vevo MicroMarker contrast agent. See Supplemental materials and methods, 4.

Contraction strength, as indicated by maximal isometric torque of the hind-limb dorsiflexors, was assessed as previously described[19] using the 1300A Whole Animal System (Aurora Scientific, Aurora, ON,

Canada). The peroneal nerve was stimulated from either side using subcutaneously inserted platinum electrodes to induce a contraction of the anterior crural muscles, and the elicited contraction was recorded. See Supplemental materials and methods, 4.

The forelimb grip-strength test was performed as previously described.[60] Mice were gently pulled by the tail after forelimb grasping a metal bar attached to a force transducer (Columbus Instruments, Columbus, OH, USA). Three sets of five consecutive grip-strength tests were recorded, each followed by a 20-min resting period. The average of the three highest values of the 15 tests was normalized to body weight. See Supplemental materials and methods, 4.

Statistical analyses

Statistical comparisons were conducted using ANOVA with mixed effects or multiple comparisons (GraphPad Prism version 7 or higher; San Diego, CA, USA). Functional animal data were analyzed using t tests. Non-linear regression methods were used to analyze the effect of log inhibitor concentration versus response.

Study approval

All *in vivo* mouse, rat, and monkey studies received prior approval from appropriate Institutional Review Boards. Pharmacokinetic studies with mice and rats were performed at WIL Research Laboratories (Ashland, OH, USA) in facilities fully accredited by the Association for Assessment and Accreditation of Laboratory Animal Care International and in accordance with the *Guide for the Care and Use of Laboratory Animals* (National Research Council, 2011). Pharmacokinetic studies with monkeys were performed at Covance (Madison, WI, USA). Functional studies in *mdx* mice were approved by the Institutional Animal Care and Use Committee and performed at the University of Minnesota (Minneapolis, MN, USA).

SUPPLEMENTAL INFORMATION

Supplemental information can be found online at https://doi.org/10.1016/j.omtm.2021.03.013.

ACKNOWLEDGMENTS

This work was conducted in Lexington, MA, USA; Minneapolis, MN, USA; Ashland, OH, USA; Madison, WI, USA; and Zwijnaarde, Belgium. The interpretation of the data was made by the authors independently. The studies were sponsored by Shire Human Genetic Therapies, a Takeda company (Lexington, MA, USA). Medical writing support was provided by Excel Medical Affairs and funded by Shire. Editorial assistance in formatting, proofreading, copyediting, and fact checking also was provided by Excel Medical Affairs. Shire Human Genetic Therapies, a Takeda company, provided funding to Excel Medical Affairs for support in writing and editing this manuscript.

AUTHOR CONTRIBUTIONS

J.B. performed experiments and was the cell biology lead for the program. Z.Z., S. Gabriëls, M.V., N.L., D.M.L., Y.H., E.B., and M.M. performed experiments, analyzed samples, and/or conducted analyses to characterize the mAbs. B.K.M., S. Gu, and M.J.T. were involved in development, engineering, and/or purification of the mAbs. M.V., S.J., and A.A. proposed the sFlt-1 pathway as a target for DMD. N.C. conducted the pharmacokinetic/pharmacodynamic analyses and interpretation. N.D.J. supervised and performed experiments. C.B. supervised experiments. H.d.H. was the chief scientific officer overseeing discovery activities at argenx. H.S.D. performed statistical analyses and wrote initial drafts of the manuscript. D.K. was the discovery project leader for mAbs in DMD.

DECLARATION OF INTERESTS

J.B., Z.Z., N.L., B.K.M., S. Gu, D.M.L., Y.H., E.B., M.M., N.C., and H.S.D. are full-time employees of Shire Human Genetic Therapies, a Takeda company, and own stock/options in Takeda. S.J., M.J.T., and D.K. were employees of Shire Human Genetic Therapies, a Takeda company, at the time of these studies. S. Gabriëls, N.D.J., C.B., and H.d.H. are full-time employees of and own stock/options in argenx. A.A. received research support from Shire Human Genetic Therapies. M.V. declares no competing interests.

REFERENCES

1. Blake, D.J., Weir, A., Newey, S.E., and Davies, K.E. (2002). Function and genetics of dystrophin and dystrophin-related proteins in muscle. Physiol. Rev. *82*, 291–329.
2. Petrof, B.J., Shrager, J.B., Stedman, H.H., Kelly, A.M., and Sweeney, H.L. (1993). Dystrophin protects the sarcolemma from stresses developed during muscle contraction. Proc. Natl. Acad. Sci. USA *90*, 3710–3714.
3. Brenman, J.E., Chao, D.S., Xia, H., Aldape, K., and Bredt, D.S. (1995). Nitric oxide synthase complexed with dystrophin and absent from skeletal muscle sarcolemma in Duchenne muscular dystrophy. Cell *82*, 743–752.
4. Thomas, G.D. (2013). Functional muscle ischemia in Duchenne and Becker muscular dystrophy. Front. Physiol. *4*, 381.
5. Ennen, J.P., Verma, M., and Asakura, A. (2013). Vascular-targeted therapies for Duchenne muscular dystrophy. Skelet. Muscle *3*, 9.
6. Shimizu-Motohashi, Y., and Asakura, A. (2014). Angiogenesis as a novel therapeutic strategy for Duchenne muscular dystrophy through decreased ischemia and increased satellite cells. Front. Physiol. *5*, 50.
7. Podkalicka, P., Mucha, O., Dulak, J., and Loboda, A. (2019). Targeting angiogenesis in Duchenne muscular dystrophy. Cell. Mol. Life Sci. *76*, 1507–1528.
8. Shibuya, M. (2013). Vascular endothelial growth factor and its receptor system: physiological functions in angiogenesis and pathological roles in various diseases. J. Biochem. *153*, 13–19.
9. Messina, S., Mazzeo, A., Bitto, A., Aguennouz, M., Migliorato, A., De Pasquale, M.G., Minutoli, L., Altavilla, D., Zentilin, L., Giacca, M., et al. (2007). VEGF overexpression via adeno-associated virus gene transfer promotes skeletal muscle regeneration and enhances muscle function in *mdx* mice. FASEB J. *21*, 3737–3746.
10. Deasy, B.M., Feduska, J.M., Payne, T.R., Li, Y., Ambrosio, F., and Huard, J. (2009). Effect of VEGF on the regenerative capacity of muscle stem cells in dystrophic skeletal muscle. Mol. Ther. *17*, 1788–1798.
11. Song, X., Zhang, Y., Hou, Z., Wu, H., Lu, S., Tang, J., Chen, X., Cui, H., Li, Y., Bi, Y., et al. (2018). Adeno-associated virus serotype 9 mediated vascular endothelial growth factor gene overexpression in mdx mice. Exp. Ther. Med. *15*, 1825–1830.
12. Gutpell, K.M., Tasevski, N., Wong, B., Hrinivich, W.T., Su, F., Hadway, J., Desjardins, L., Lee, T.Y., and Hoffman, L.M. (2017). ANG1 treatment reduces muscle pathology and prevents a decline in perfusion in DMD mice. PLoS ONE *12*, e0174315.
13. Nagy, J.A., and Senger, D.R. (2006). VEGF-A, cytoskeletal dynamics, and the pathological vascular phenotype. Exp. Cell Res. *312*, 538–548.

14. Gianni-Barrera, R., Trani, M., Fontanellaz, C., Heberer, M., Djonov, V., Hlushchuk, R., and Banfi, A. (2013). VEGF over-expression in skeletal muscle induces angiogenesis by intussusception rather than sprouting. Angiogenesis *16*, 123–136.

15. Eppler, S.M., Combs, D.L., Henry, T.D., Lopez, J.J., Ellis, S.G., Yi, J.H., Annex, B.H., McCluskey, E.R., and Zioncheck, T.F. (2002). A target-mediated model to describe the pharmacokinetics and hemodynamic effects of recombinant human vascular endothelial growth factor in humans. Clin. Pharmacol. Ther. *72*, 20–32.

16. Kendall, R.L., and Thomas, K.A. (1993). Inhibition of vascular endothelial cell growth factor activity by an endogenously encoded soluble receptor. Proc. Natl. Acad. Sci. USA *90*, 10705–10709.

17. Ikeda, T., Sun, L., Tsuruoka, N., Ishigaki, Y., Yoshitomi, Y., Yoshitake, Y., and Yonekura, H. (2011). Hypoxia down-regulates sFlt-1 (sVEGFR-1) expression in human microvascular endothelial cells by a mechanism involving mRNA alternative processing. Biochem. J. *436*, 399–407.

18. Fan, X., Rai, A., Kambham, N., Sung, J.F., Singh, N., Petitt, M., Dhal, S., Agrawal, R., Sutton, R.E., Druzin, M.L., et al. (2014). Endometrial VEGF induces placental sFLT1 and leads to pregnancy complications. J. Clin. Invest. *124*, 4941–4952.

19. Verma, M., Asakura, Y., Hirai, H., Watanabe, S., Tastad, C., Fong, G.H., Ema, M., Call, J.A., Lowe, D.A., and Asakura, A. (2010). *Flt-1* haploinsufficiency ameliorates muscular dystrophy phenotype by developmentally increased vasculature in *mdx* mice. Hum. Mol. Genet. *19*, 4145–4159.

20. Verma, M., Shimizu-Motohashi, Y., Asakura, Y., Ennen, J.P., Bosco, J., Zhou, Z., Fong, G.H., Josiah, S., Keefe, D., and Asakura, A. (2019). Inhibition of FLT1 ameliorates muscular dystrophy phenotype by increased vasculature in a mouse model of Duchenne muscular dystrophy. PLoS Genet. *15*, e1008468.

21. Manning, J., and O'Malley, D. (2015). What has the mdx mouse model of Duchenne muscular dystrophy contributed to our understanding of this disease? J. Muscle Res. Cell Motil. *36*, 155–167.

22. Klarenbeek, A., Blanchetot, C., Schragel, G., Sadi, A.S., Ongenae, N., Hemrika, W., Wijdenes, J., Spinelli, S., Desmyter, A., Cambillau, C., et al. (2016). Combining somatic mutations present in different in vivo affinity-matured antibodies isolated from immunized Lama glama yields ultra-potent antibody therapeutics. Protein Eng. Des. Sel. *29*, 123–133.

23. Könning, D., Zielonka, S., Grzeschik, J., Empting, M., Valldorf, B., Krah, S., Schröter, C., Sellmann, C., Hock, B., and Kolmar, H. (2017). Camelid and shark single domain antibodies: structural features and therapeutic potential. Curr. Opin. Struct. Biol. *45*, 10–16.

24. Hamers-Casterman, C., Atarhouch, T., Muyldermans, S., Robinson, G., Hamers, C., Songa, E.B., Bendahman, N., and Hamers, R. (1993). Naturally occurring antibodies devoid of light chains. Nature *363*, 446–448.

25. Cuende, J., Liénart, S., Dedobbeleer, O., van der Woning, B., De Boeck, G., Stockis, J., Huygens, C., Colau, D., Somja, J., Delvenne, P., et al. (2015). Monoclonal antibodies against GARP/TGF-β1 complexes inhibit the immunosuppressive activity of human regulatory T cells in vivo. Sci. Transl. Med. *7*, 284ra56.

26. Markovic-Mueller, S., Stuttfeld, E., Asthana, M., Weinert, T., Bliven, S., Goldie, K.N., Kisko, K., Capitani, G., and Ballmer-Hofer, K. (2017). Structure of the full-length VEGFR-1 extracellular domain in complex with VEGF-A. Structure *25*, 341–352.

27. Wines, B.D., Powell, M.S., Parren, P.W., Barnes, N., and Hogarth, P.M. (2000). The IgG Fc contains distinct Fc receptor (FcR) binding sites: the leukocyte receptors Fc γ RI and Fc γ RIIa bind to a region in the Fc distinct from that recognized by neonatal FcR and protein A. J. Immunol. *164*, 5313–5318.

28. Birnkrant, D.J., Bushby, K., Bann, C.M., Apkon, S.D., Blackwell, A., Brumbaugh, D., Case, L.E., Clemens, P.R., Hadjiyannakis, S., Pandya, S., et al.; DMD Care Considerations Working Group (2018). Diagnosis and management of Duchenne muscular dystrophy, part 1: diagnosis, and neuromuscular, rehabilitation, endocrine, and gastrointestinal and nutritional management. Lancet Neurol. *17*, 251–267.

29. Shieh, P.B. (2018). Emerging strategies in the treatment of Duchenne muscular dystrophy. Neurotherapeutics *15*, 840–848.

30. Shimizu-Motohashi, Y., Komaki, H., Motohashi, N., Takeda, S., Yokota, T., and Aoki, Y. (2019). Restoring dystrophin expression in Duchenne muscular dystrophy: current status of therapeutic approaches. J. Pers. Med. *9*, 1.

31. Sander, M., Chavoshan, B., Harris, S.A., Iannaccone, S.T., Stull, J.T., Thomas, G.D., and Victor, R.G. (2000). Functional muscle ischemia in neuronal nitric oxide synthase-deficient skeletal muscle of children with Duchenne muscular dystrophy. Proc. Natl. Acad. Sci. USA *97*, 13818–13823.

32. Turanov, A.A., Lo, A., Hassler, M.R., Makris, A., Ashar-Patel, A., Alterman, J.F., Coles, A.H., Haraszti, R.A., Roux, L., Godinho, B.M.D.C., et al. (2018). RNAi modulation of placental sFLT1 for the treatment of preeclampsia. Nat. Biotechnol. *36*, 1164–1173.

33. Gatta, A.K., Hariharapura, R.C., Udupa, N., Reddy, M.S., and Josyula, V.R. (2018). Strategies for improving the specificity of siRNAs for enhanced therapeutic potential. Expert Opin. Drug Discov. *13*, 709–725.

34. Chatterjee, S., Heukamp, L.C., Siobal, M., Schöttle, J., Wieczorek, C., Peifer, M., Frasca, D., Koker, M., König, K., Meder, L., et al. (2013). Tumor VEGF:VEGFR2 autocrine feed-forward loop triggers angiogenesis in lung cancer. J. Clin. Invest. *123*, 1732–1740.

35. Matsumoto, T., and Claesson-Welsh, L. (2001). VEGF receptor signal transduction. Sci. STKE *2001*, re21.

36. Hoeben, A., Landuyt, B., Highley, M.S., Wildiers, H., Van Oosterom, A.T., and De Bruijn, E.A. (2004). Vascular endothelial growth factor and angiogenesis. Pharmacol. Rev. *56*, 549–580.

37. Verma, M., Asakura, Y., Murakonda, B.S.R., Pengo, T., Latroche, C., Chazaud, B., McLoon, L.K., and Asakura, A. (2018). Muscle satellite cell cross-talk with a vascular niche maintains quiescence via VEGF and notch signaling. Cell Stem Cell *23*, 530–543.e9.

38. Hasan, J., Beharry, K.D., Valencia, A.M., Strauss, A., and Modanlou, H.D. (2009). Soluble vascular endothelial growth factor receptor 1 in tracheal aspirate fluid of preterm neonates at birth may be predictive of bronchopulmonary dysplasia/chronic lung disease. Pediatrics *123*, 1541–1547.

39. Boutsikou, T., Malamitsi-Puchner, A., Economou, E., Boutsikou, M., Puchner, K.P., and Hassiakos, D. (2006). Soluble vascular endothelial growth factor receptor-1 in intrauterine growth restricted fetuses and neonates. Early Hum. Dev. *82*, 235–239.

40. Lammerts van Bueren, J.J., Bleeker, W.K., Bøgh, H.O., Houtkamp, M., Schuurman, J., van de Winkel, J.G., and Parren, P.W. (2006). Effect of target dynamics on pharmacokinetics of a novel therapeutic antibody against the epidermal growth factor receptor: implications for the mechanisms of action. Cancer Res. *66*, 7630–7638.

41. Yen, P., Finley, S.D., Engel-Stefanini, M.O., and Popel, A.S. (2011). A two-compartment model of VEGF distribution in the mouse. PLoS ONE *6*, e27514.

42. Clegg, L.E., and Mac Gabhann, F. (2017). A computational analysis of in vivo VEGFR activation by multiple co-expressed ligands. PLoS Comput. Biol. *13*, e1005445.

43. Ryman, J.T., and Meibohm, B. (2017). Pharmacokinetics of monoclonal antibodies. CPT Pharmacometrics Syst. Pharmacol. *6*, 576–588.

44. Walker, K.W., Salimi-Moosavi, H., Arnold, G.E., Chen, Q., Soto, M., Jacobsen, F.W., and Hui, J. (2019). Pharmacokinetic comparison of a diverse panel of non-targeting human antibodies as matched IgG1 and IgG2 isotypes in rodents and non-human primates. PLoS ONE *14*, e0217061.

45. Brissova, M., Shostak, A., Fligner, C.L., Revetta, F.L., Washington, M.K., Powers, A.C., and Hull, R.L. (2015). Human islets have fewer blood vessels than mouse islets and the density of islet vascular structures is increased in type 2 diabetes. J. Histochem. Cytochem. *63*, 637–645.

46. Pautu, V., Mellinger, A., Resnier, P., Lepeltier, E., Martin, L., Boussemart, L., Letournel, F., Passirani, C., and Clere, N. (2019). Melanoma tumour vasculature heterogeneity: from mice models to human. J. Cancer Res. Clin. Oncol. *145*, 589–597.

47. Imoukhuede, P.I., and Popel, A.S. (2012). Expression of VEGF receptors on endothelial cells in mouse skeletal muscle. PLoS ONE *7*, e44791.

48. Kho, A.T., Kang, P.B., Kohane, I.S., and Kunkel, L.M. (2006). Transcriptome-scale similarities between mouse and human skeletal muscles with normal and myopathic phenotypes. BMC Musculoskelet. Disord. *7*, 23.

49. Klingler, W., Jurkat-Rott, K., Lehmann-Horn, F., and Schleip, R. (2012). The role of fibrosis in Duchenne muscular dystrophy. Acta Myol. *31*, 184–195.

50. Im, W.B., Phelps, S.F., Copen, E.H., Adams, E.G., Slightom, J.L., and Chamberlain, J.S. (1996). Differential expression of dystrophin isoforms in strains of *mdx* mice with different mutations. Hum. Mol. Genet. *5*, 1149–1153.

51. Beastrom, N., Lu, H., Macke, A., Canan, B.D., Johnson, E.K., Penton, C.M., Kaspar, B.K., Rodino-Klapac, L.R., Zhou, L., Janssen, P.M.L., and Montanaro, F. (2011).

mdx(^{5}cv) mice manifest more severe muscle dysfunction and diaphragm force deficits than do mdx Mice. Am. J. Pathol. *179*, 2464–2474.

52. Kobayashi, Y.M., Rader, E.P., Crawford, R.W., Iyengar, N.K., Thedens, D.R., Faulkner, J.A., Parikh, S.V., Weiss, R.M., Chamberlain, J.S., Moore, S.A., and Campbell, K.P. (2008). Sarcolemma-localized nNOS is required to maintain activity after mild exercise. Nature *456*, 511–515.

53. Huang, P.L., Dawson, T.M., Bredt, D.S., Snyder, S.H., and Fishman, M.C. (1993). Targeted disruption of the neuronal nitric oxide synthase gene. Cell *75*, 1273–1286.

54. Wehling-Henricks, M., Jordan, M.C., Roos, K.P., Deng, B., and Tidball, J.G. (2005). Cardiomyopathy in dystrophin-deficient hearts is prevented by expression of a neuronal nitric oxide synthase transgene in the myocardium. Hum. Mol. Genet. *14*, 1921–1933.

55. Asai, A., Sahani, N., Kaneki, M., Ouchi, Y., Martyn, J.A., and Yasuhara, S.E. (2007). Primary role of functional ischemia, quantitative evidence for the two-hit mechanism, and phosphodiesterase-5 inhibitor therapy in mouse muscular dystrophy. PLoS ONE *2*, e806.

56. Gregorevic, P., Blankinship, M.J., Allen, J.M., Crawford, R.W., Meuse, L., Miller, D.G., Russell, D.W., and Chamberlain, J.S. (2004). Systemic delivery of genes to striated muscles using adeno-associated viral vectors. Nat. Med. *10*, 828–834.

57. Wagner, K.R., Wong, B.L., Byrne, B.J., Tian, C., Jacobsen, L.K., Tirucherai, G.S., Rabbia, M., Kletzl, H., Dukart, J., Ong, R., et al. (2019). A phase 1b/2 study of the anti-myostatin adnectin RG6206 (BMS-986089) in ambulatory boys with Duchenne muscular dystrophy: a 72-week treatment update. Neurology *92*, P1.6-062.

58. Marraffino, S., Binks, M., Palmer, J., Sherlock, S., Purohit, V., and Charnas, L. (2019). A phase 2 randomized placebo-control trial of domagrozumab in boys with Duchenne muscular dystrophy (DMD). Neuromuscul. Disord. *29*, S167.

59. Wallace, B., Peisl, A., Seedorf, G., Nowlin, T., Kim, C., Bosco, J., Kenniston, J., Keefe, D., and Abman, S.H. (2018). Anti-sFlt-1 therapy preserves lung alveolar and vascular growth in antenatal models of bronchopulmonary dysplasia. Am. J. Respir. Crit. Care Med. *197*, 776–787.

60. Aartsma-Rus, A., and van Putten, M. (2014). Assessing functional performance in the *mdx* mouse model. J. Vis. Exp. 51303.

OMTM, Volume *21*

Supplemental information

VEGFR-1/Flt-1 inhibition increases angiogenesis and improves muscle function in a mouse model of Duchenne muscular dystrophy

Jennifer Bosco, Zhiwei Zhou, Sofie Gabriëls, Mayank Verma, Nan Liu, Brian K. Miller, Sheng Gu, Dianna M. Lundberg, Yan Huang, Eilish Brown, Serene Josiah, Muthuraman Meiyappan, Matthew J. Traylor, Nancy Chen, Atsushi Asakura, Natalie De Jonge, Christophe Blanchetot, Hans de Haard, Heather S. Duffy, and Dennis Keefe

1. Antibody Purification

In order to obtain pure antibody (Ab) samples for animal experiments, Chinese hamster ovary (CHO) Epstein-Barr nuclear antigen-1 (EBNA) cells were electroporated with anti-human Flt-1 full IgG lead candidates using the MaxCyte STX system (MaxCyte, Gaithersburg, MD). Two hundred µg/mL anti-human Flt-1 ATUM vector pD2610-v5 DNA and 50 µg/mL XBP1s DNA were electroporated into CHO EBNA cells using MaxCyte 2.1 (10 mL electroporation volume with 400×10^9 cells). After transfection, cells were transferred to a 1-L flask with EX-Cell Advanced CHO Fed-batch Medium (Sigma-Aldrich) for recovery at 37°C. At 24 h after transfection, 3% EX-CELL Advanced CHO Feed 1 (with glucose) (AF1; Sigma-Aldrich, St. Louis, MO) was added and cells were transferred to 33°C. At 48 h after transfection, 3% AF1 and 0.125% N,N-dimethylacetamide were added; 3% AF1 was continued for 10 to 14 days or until viability dropped below 75%. Culture media was collected and frozen at –80°C for purification.

For purification, culture media was thawed and loaded onto an equilibrated MabSelect SuRe column (GE Healthcare Life Sciences) at 20 mg/mL resin. The column was washed with 5 volumes of phosphate-buffered saline (PBS) pH 7. 2 (Invitrogen) and then eluted with 10 volumes of 100 mM glycine pH 3 (Sigma) and finally neutralized with a 50:1 ratio of 1 M Tris hydrochloride (HCl) pH 9. Fractions were pooled and dialyzed overnight at 4ºC in 40 mM Tris HCl pH 8.1 and loaded onto an equilibrated HiTrap Q HP column (GE Healthcare Life Sciences) at 100 mg/mL resin. The column was washed with 10 volumes of 40 mm Tris HCl pH 8.1 and then eluted with 5 volumes of PBS, 1 M sodium chloride. Fractions were pooled and dialyzed into PBS.

2. Pharmacokinetics of 21B3 and 27H6 in Mice, Rats, and Monkeys

Measurement of 21B3 in Mice

Male CD-1 mice (n = 27, Charles River Laboratories, Wilmington, MA) received a single intravenous (i.v.) bolus dose of 10 mg/kg [^{125}I]-21B3. At 0.083, 0.25, and 4 h, and 5, 21, and 28 days after dosing, a blood sample was collected from 3 mice per dose group. At 7 time points after dosing (0.5, 1, 8, and 24 h, and 3, 7, and 14 days), 3 mice per dose group were euthanized and blood, diaphragm, and tibialis samples were collected. Serum samples were precipitated with trichloroacetic acid and centrifuged to precipitate serum proteins; the supernatant fractions and pellets were analyzed. All serum and muscle samples were analyzed for total radioactivity using gamma counting techniques.

Measurement of 27H6 in Mice

Male CD1 mice (n = 42 mice per dose group) received a single i.v. bolus dose of 0.3, 3, or 30 mg/kg [^{125}I]-27H6. At 14 time points after dosing (5, 15, and 30 min; 1, 2, 4, 8, and 24 h; and 3, 5, 7, 14, 18, and 28 days) a terminal blood sample was collected from 3 mice per dose group. To determine the fraction of [^{125}I] that was unbound to 27H6 in each sample, serum samples were precipitated with trichloroacetic acid and centrifuged to precipitate proteins; the supernatant fraction and pellet were analyzed for total radioactivity using gamma counting techniques.

Measurement of 27H6 in Rats

Male Sprague Dawley rats (n = 24 rats per dose group, Charles River Laboratories) received a single i.v. bolus dose of 0.3, 3, or 30 mg/kg 27H6 h(D/A) LALA. Before dose and at 16 time points after dosing (5, 15, and 30 min; 1, 2, 4, 8, and 24 h; and 3, 5, 7, 10, 14, 18, 24, and 28 days), serial blood samples were collected from 3 rats per dose group. 27H6 h(D/A) LALA was measured using an enzyme-linked immunosorbent assay (ELISA).

Measurement of 27H6 in Monkeys

Three dose groups, each consisting of 1 male and 1 female cynomolgus monkey (Covance), received a single i.v. bolus of 0.3, 3, or 30 mg/kg 27H6. Before dose and at 16 time points after dosing (5 min; 1, 2, 6, and 24 h; and 2, 3, 4, 5, 7, 10, 14, 21, 28, 35, and 45 days), serial blood samples were collected and analyzed using an anti–sFlt-1 detection ELISA.

3. Histopathology Analysis

All immunohistochemistry staining was performed on 5 µM paraffin-embedded sections of the diaphragm or tibialis muscles. For CD31 staining, goat anti-CD31/PECAM-1 Ab (R&D Systems, AF3628, 1:50) was used as the primary Ab and isotype IgG as a negative control; biotin-labeled rabbit anti-goat IgG (Vector Laboratories) and ABC Kit (Vector Laboratories) were applied as the detection system and the positive signal was revealed with 3,3′-diaminobenzidine. For detection of fibrosis, rabbit anti-collagen I Ab (Boster Bio, PA2140-2, 1:1000) was used.

BOND Polymer Refine kit (Leica Biosystems, DS9800) was applied as the detection system. The positive cells were identified as brown in color and nuclei were stained blue. The stained slides were scanned with Aperio AT2 scanner (Leica Biosystems). The whole digital slides were viewed and analyzed by Aperio ImageScope (Leica Biosystems). The positive pixel count algorithm was selected and adjusted to cover each individual positive staining for analysis. The data were presented as positivity, which was obtained from the following formula: positivity (%) = positive area (pixels) / total stain area (pixels) × 100%.

4. Measurement of Muscle Perfusion and Muscle Function

Red blood cell flux was measured using a laser Doppler flowmeter with an MP7a probe as previously described,[1] according to the manufacturer's instructions (Moor Instruments). Fur from the right hind leg was removed using a chemical depilatory. Flux was measured at ≥ 10 different spots on the tibialis anterior muscle. An arbitrary unit was determined as the average arbitrary unit value during a plateau phase of each measurement.

Microbubble angiography was performed using the Vevo 2100 Imaging System with Vevo MicroMarker contrast agent (Fujifilm). Mice were sedated, shaved, and depilated over the medial hind limb. Mice were sedated using 4% isoflurane and secured to a heated platform using tape. Pre-warmed ultrasound gel was applied to the probe and the muscle was visualized using B-mode and Doppler ultrasound to locate the femoral artery/vein; the region of interest of exact same size was drawn for the muscle around the vessels. Contrast agent (60 µL containing 2×10^9 bubbles/mL) was administered over 2 s retro-orbitally and non-linear contrast imaging was performed. The peak enhancement was measured using Vevo 2100 software (Fujifilm).

To measure contraction strength, mice were anesthetized with a cocktail of fentanyl citrate (10 mg/kg body weight), droperidol (0.2 mg/kg body weight), and diazepam (5 mg/kg body weight). The left hind limb of each mouse was shaved and aseptically prepared, and each mouse was positioned on a heated platform with its left foot placed on a metal foot plate attached to the shaft of a servomotor. Two platinum electrodes (model E2-12, Grass Technologies) were inserted subcutaneously on either side of the peroneal nerve. A stimulator and stimulus isolation unit stimulated the peroneal nerve via the platinum electrodes to induce a contraction of the anterior crural muscles. For whole muscle contraction, the anterior crural muscles of untreated and 21B3-treated mice were stimulated and the contraction elicited was recorded. The current was adjusted to elicit the maximum twitch and adjusted to 15% additional current for the isometric contraction. The parameters for stimulation were set at a 200 ms contraction duration consisting of 0.5 ms square-wave pulses at 250 Hz. Torque was normalized according to body weight.

The forelimb grip strength test was performed as previously described.[2] Mice were gently pulled by the tail after forelimb-grasping a metal bar attached to a force transducer (Columbus Instruments). Grip strength tests were performed by the same blinded examiner. Five consecutive grip strength tests were recorded, then the mice were returned to the cage for a resting period of 20 min. Three series of pulls were then performed, each followed by a 20-min resting period. The average of the three highest values of the 15 collected values was normalized to the body weight for comparison.

Table S1. Antibody binding to VEGFR-1, VEGFR-2, and VEGFR-3

Example binding affinity of mAbs (50 μg/mL single injection) was measured using a Biacore method (direct coating antigen). Binding to VEGFR-2 and VEGFR-3 was not observed, even at high doses of antibody, indicating that there is no cross-reactivity to these isoforms.

mAb	VEGFR-1	VEGFR-2	VEGFR-3
Blank	2.91	3.64	4.45
13B4	**92.6**	2.75	3
21C6	**181**	1.74	3.25
21B3	**78.7**	3.32	4.26

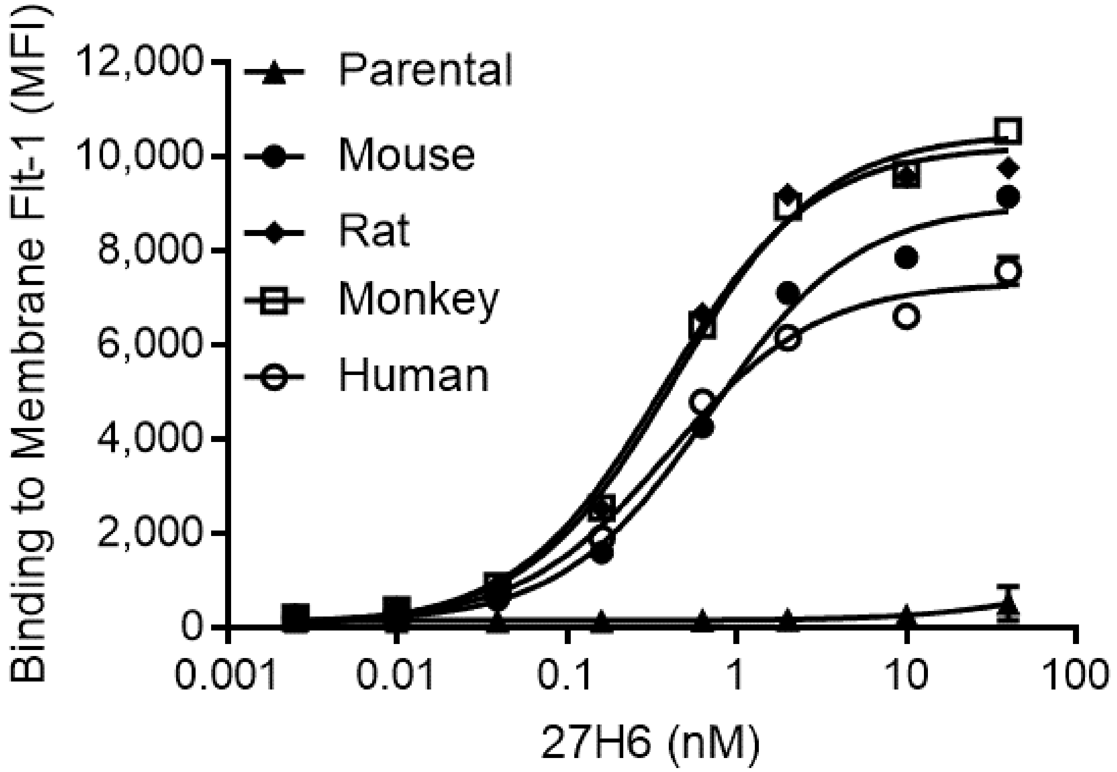

Figure S1. AF647-Labeled 27H6 Binding to Membrane-Bound Flt-1 in HEK293 Cells Overexpressing Mouse, Rat, Monkey, and Human sFlt-1

To assess species-specific binding, 27H6 and a control antibody (Ab) (ZMAB – Human IgG1, AB Biosciences, Concord, MA) were labelled with AF467 (Invitrogen, Waltham, MA) and various concentrations were added to HEK293 cells (ATCC) overexpressing species-specific sFlt-1 (n = 2 for each species). 250,000 cells were plated and incubated overnight. 1 µL of various concentrations of 27H6-AF647 or control antibody was added to the wells, followed by a 15-min incubation at 4ºC. The cells were washed and fixed in 2% paraformaldehyde for 10 min at 4ºC. The samples were analyzed using a FACSCanto II flow cytometer (BD Biosciences, San Jose, CA). Untransfected cells were used as the parental control. MFI, mean fluorescence intensity.

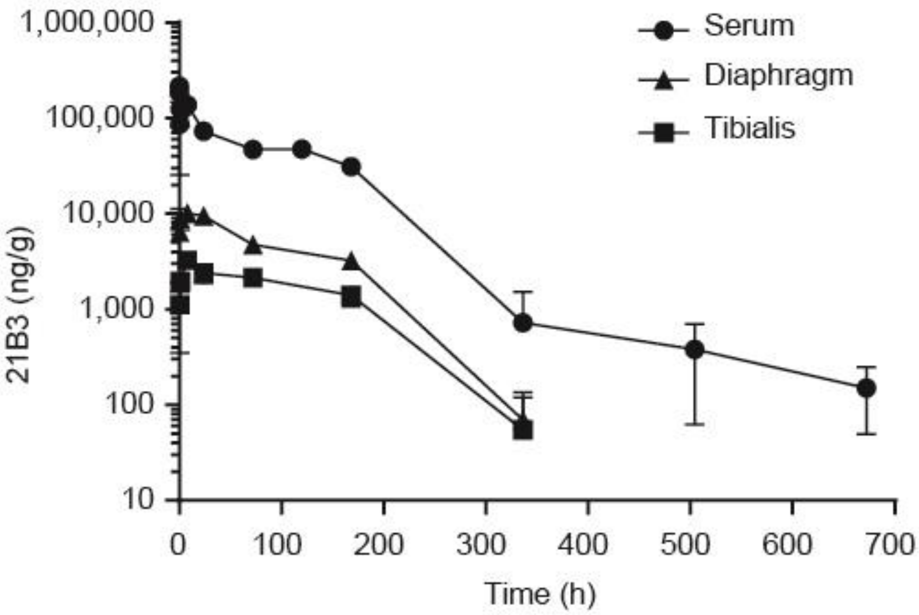

Figure S2. Mean Concentrations of [125I]-21B3 in Serum and in Diaphragm and Tibialis Anterior Muscles of Mice

Mice were administered a single 10 mg/kg intravenous bolus dose of [^{125}I]-21B3 in the serum and skeletal muscles. Concentration-time profiles show mean ± standard deviation of n = 3 mice at each time point. Values represent test article equivalents.

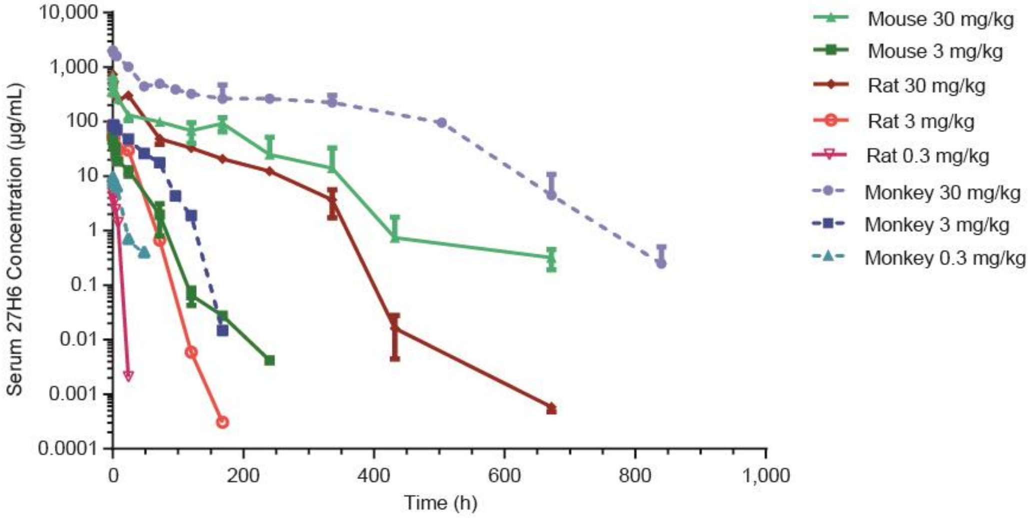

Figure S3. Mean Serum Antibody Concentrations in Mice, Rats, and Monkeys After a Single Intravenous Bolus Injection of 27H6

Rats and cynomolgus monkeys were administered a single intravenous bolus dose of 0.3, 3, or 30 mg/kg 27H6. Blood samples were collected up to 28 days after dosing. Levels of 27H6 were measured using an anti–Flt-1 detection enzyme-linked immunosorbent assay. Concentration-time profiles show mean ± standard deviation of n = 3 mice, n = 2 rats, and n = 2 monkeys at each time point. There was a non-parallel terminal clearance slope across different dose levels (0.3 to 30 mg/kg) with higher clearance at lower doses. Target-mediated disposition occurred when drug exposure was lower than target Flt-1 concentrations.

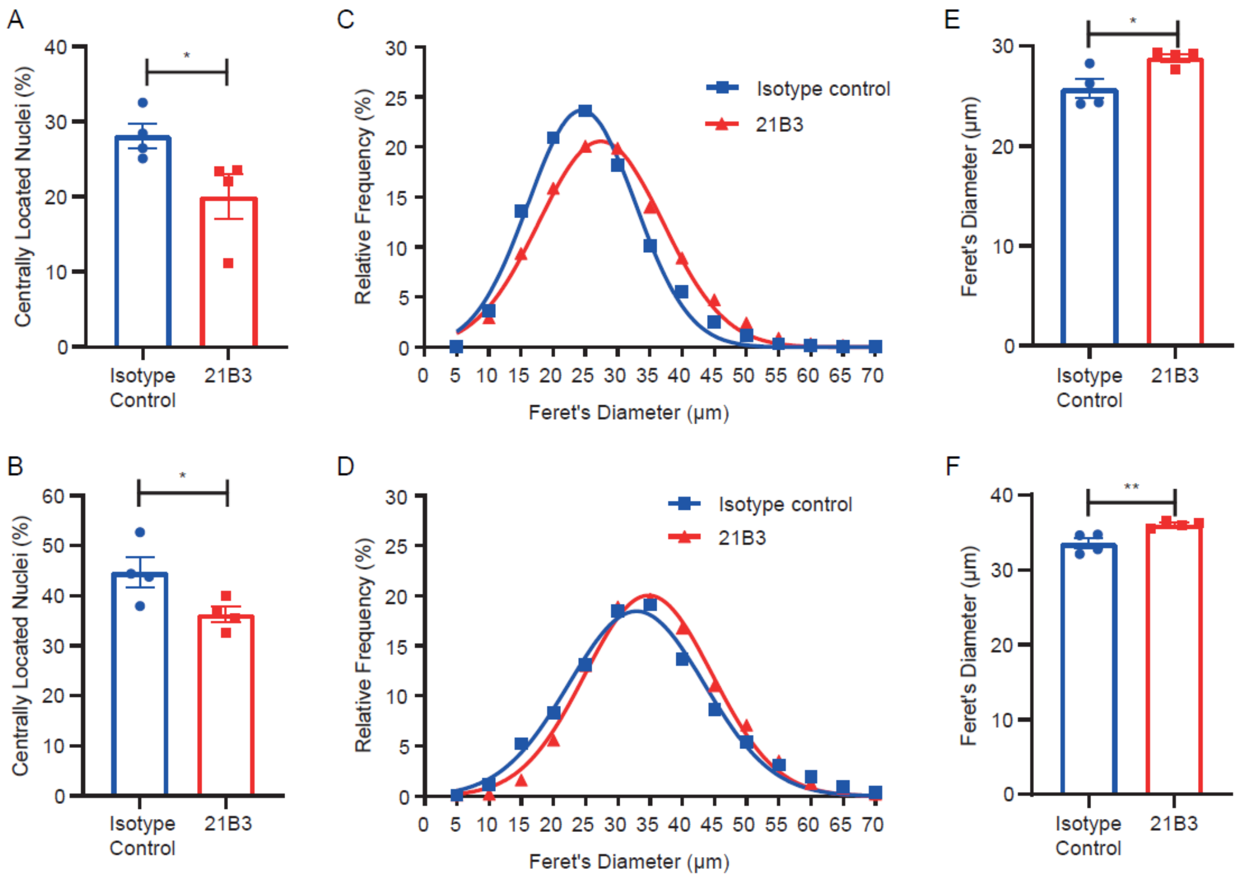

Figure S4. Administration of 21B3 improves muscle histology and function in mdx mice.
(A, B) Diaphragm and tibialis anterior muscle fiber turnover is reduced in *mdx* mice administrated 20 mg/kg 21B3 twice weekly for 4 weeks compared with controls (n = 4 per dose group) as evaluated by centrally located nuclei. (C, D) Distributions and (E, F) averages of mean fiber diameter in diaphragm and tibialis anterior muscle of *mdx* mice administrated 21B3 or control. $*p < 0.05$, $**p < 0.01$ using t tests.

Histological analysis of diaphragm (A, C, E) and tibialis anterior (B, D, F) muscle of *mdx* mice (9–10 weeks old) administered 20 mg/kg 21B3 i.v. twice weekly for 4 weeks. Tissues were frozen fresh using LiN_2 chilled isopentane and stored at –80ºC. Transverse cryosections (8 μm thick) were used for all histological analyses. Hematoxylin & eosin staining were performed as previously described[3]. Microscopic images were captured by a DP-1 digital camera attached to a BX51 fluorescence microscope with 10 × or 40 × UPlanFL N objectives (all from Olympus). Image processing and manual enumeration of the fiber diameter was conducted using Fiji.[4]

References

1. Verma, M, Asakura, Y, Hirai, H, Watanabe, S, Tastad, C, Fong, GH, Ema, M, Call, JA, Lowe, DA, and Asakura, A (2010). *Flt-1* haploinsufficiency ameliorates muscular dystrophy phenotype by developmentally increased vasculature in *mdx* mice. *Hum Mol Genet* **19**: 4145-4159.

2. Aartsma-Rus, A, and van Putten, M (2014). Assessing functional performance in the *mdx* mouse model. *J Vis Exp* **85**: 51303.

3. Verma, M, Shimizu-Motohashi, Y, Asakura, Y, Ennen, JP, Bosco, J, Zhou, Z, Fong, GH, Josiah, S, Keefe, D, and Asakura, A (2019). Inhibition of FLT1 ameliorates muscular dystrophy phenotype by increased vasculature in a mouse model of Duchenne muscular dystrophy. *PLoS Genet* **15**: e1008468.

4. Schindelin J, Arganda-Carreras I, Frise E, Kaynig V, Longair M, Pietzsch T, Preibisch, S, Rueden, C, Saalfeld, S, Schmid, B, *et al*. (2012) Fiji: an open-source platform for biological-image analysis. *Nat Methods* **9**: 676–682.
